# Coherent Mixing of Singlet and Triplet States in Acrolein and Ketene: A Computational Strategy for Simulating the Electron–Nuclear Dynamics of Intersystem Crossing

**DOI:** 10.1021/acs.jpclett.3c01187

**Published:** 2023-06-26

**Authors:** Don Danilov, Andrew J Jenkins, Michael J Bearpark, Graham A Worth, Michael A Robb

**Affiliations:** † Department of Chemistry, University College London, 20 Gordon St., WC1H 0AJ London, United Kingdom; ‡ Department of Chemistry, Imperial College London, Molecular Sciences Research Hub, 82 Wood Lane, W12 0BZ London, United Kingdom; § Department of Chemistry, University of Washington, Seattle, Washington 98195, United States

## Abstract

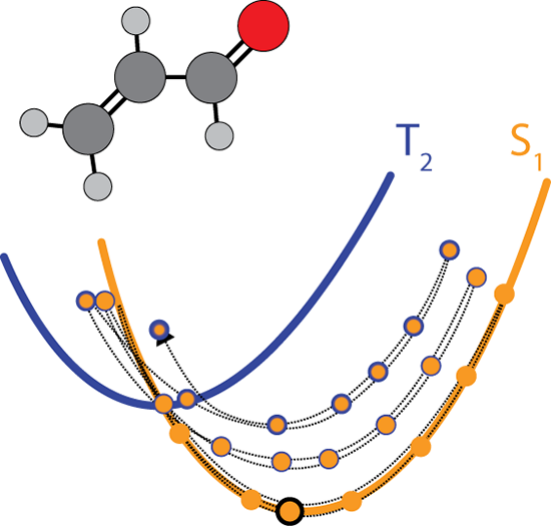

We present a theoretical study of intersystem crossing (ISC) in acrolein and ketene with the Ehrenfest method that can describe a superposition of singlet and triplet states. Our simulations illustrate a new mechanistic effect of ISC, namely, that a superposition of singlets and triplets yields nonadiabatic dynamics characteristic of that superposition rather than the constituent state potential energy surfaces. This effect is particularly significant in ketene, where mixing of singlet and triplet states along the approach to a singlet/singlet conical intersection occurs, with the spin–orbit coupling (SOC) remaining small throughout. In both cases, the effects require many recrossings of the singlet/triplet state crossing seam, consistent with the textbook treatment of ISC.

Our focus in this article is the description of coupled electron–nuclear dynamics in molecules with spin–orbit coupling induced superpositions of electronic states of different spin multiplicities. This is analogous to attochemistry,^1−3^ where the superpositions are created in laser experiments.

In photochemical dynamics, radiationless decay—internal conversion (IC) between states of same spin multiplicity and intersystem crossing^4−6^ (ISC) between states of different spin multiplicity—is conventionally described in terms of descent through electronic states linked by potential energy surface crossings/conical intersections.^7−11^.^12,13^ Many nonadiabatic dynamics methods, e.g., surface hop, describe this effect by moving on one electronic state surface at a time.^6,14^

Here we explore an alternative formalism^15^ where radiationless transitions are continuous and take place on a single time-dependent potential energy surface as opposed to being discontinuous (e.g. via a surface hop) at a crossing. Thus, a singlet + triplet state superposition will generate a gradient for nuclei that is different from either the singlet or triplet alone (and that will evolve with time), and the subsequent nuclear motion will be determined by the mixed state. At the start of the dynamics, in addition to the intrastate gradient components, there is an interstate gradient component that arises from the mixing. At later times, there is an instantaneous gradient that arises from the electron dynamics (as demonstrated before in the glycine^16^ and benzene^17^ cations).

Intersystem crossing in photochemistry is known to be relatively slow.^18^ In this work, we will see this effect as a slow buildup of a mixed singlet + triplet state after repeated passage across the crossing region. The reaction path for ISC will therefore be a time-dependent mixture of singlet and triplet states determined by the solution of the time-dependent Schrodinger equation (TDSE).

We now discuss the formalism employed; namely, the 1 electron average spin–orbit coupling Hamiltonian and how this is included in an Ehrenfest dynamics framework.

Spin–orbit coupling (SOC) mixes electronic states with different spin multiplicities. In the context of photochemical dynamics, SOC leads to intersystem crossing (ISC), and the dynamics of the ISC is expected to be governed by the size of the SOC between the states involved. SOC is a relativistic effect^19^ whose contribution can be approximated by the so-called Breit–Pauli term,^20^ and contribution to the molecular Hamiltonian in atomic units can be expressed as
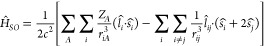
1where the nuclei are indexed with capital letters and the electrons with lowercase letters, *Z*_*A*_ is the charge on nucleus *A*, *r*_*iA*_ is the distance between nucleus *A* and electron *i*, *r*_*ij*_ is the interelectron distance between electrons *i* and *j*, *l̂* is the orbital angular momentum operator, *Ŝ* is the spin operator, and *c* is the speed of light.

To avoid the computationally expensive two-electron term we use the approximation of Koseki et al.^21,22^ wherein the two-electron term is incorporated into the one-electron term using an empirical effective nuclear charge (*Z*_*A*,*eff*_) defined in ref (21):
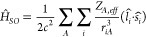
2SOC also acts to break the degeneracy of multiplet microstates (doublets, triplets, *etc.*) (for example, the well documented splitting of the sodium ^2^*P* state into ^2^*P*_1/2_ and ^2^*P*_3/2_ by approximately 2 meV.^6^ This effect is relatively small for light atoms, and, as is common practice,we treat the multiplet microstates (e.g., *m*_*s*_ = −1, 0, +1 in a triplet) as one “average” state for the purposes of dynamics.^23−26^ In this work we employ this averaged triplet approximation and test it by evaluating the triplet splitting at points of high singlet/triplet mixing along the trajectories using a relativistic calculation^27^ in the SI.

In our dynamics simulation, we use the Ehrenfest method^15,28−31^ which describes the electronic wave function in a Complete Active Space Configuration Interaction (CAS-CI) formalism. The Ehrenfest electronic state is expressed as a linear combination on the basis of all Slater determinants formed in the active space with zero *z* spin component (so that both singlet and triplet states can be represented). A related approach is exact factorization,^32,33^ which can also be adapted to include ISC as has been described by Talotta et al.^34^

In this article we will examine the intersystem crossing dynamics for acrolein and ketene (shown in Scheme 1) which exhibit different mechanistic effects of ISC. The potential energy surface topology of these simple organic carbonyl–allylic systems has been well characterized with a wide range of other electronic structure and dynamics methods.^26,35−45^ In acrolein, the spatial/orbital configurations of singlet and triplet are different, whereas in ketene they are the same (Scheme 1). As a result, we have a large difference in spin–orbit couplings: ∼65 cm^–1^ in acrolein versus ∼0.1 cm^–1^ in ketene at the S_1_ minima. According to El-Sayed’s rule^46^ we should therefore expect the yield of triplet in acrolein to be greater.

**Scheme 1:**
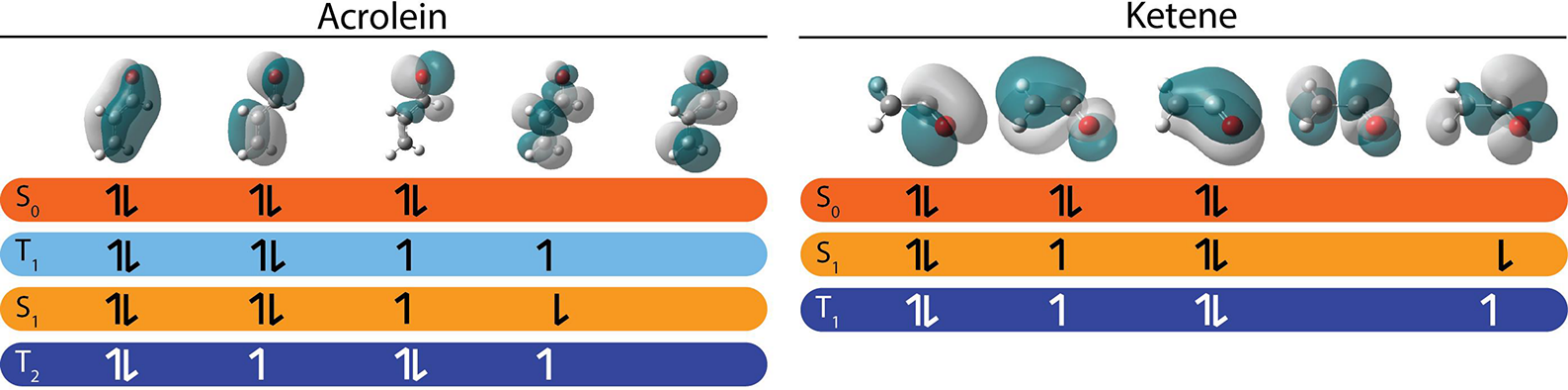
Electronic States and Their Dominant Configurations from a SA-CASSCF(6,5)/6-31G* Calculation at the S_1_ Minimum Geometry for Acrolein and Ketene We illustrate the M_s_ = 1 configuration of the triplets for visual clarity (in the dynamics we use the M_s_ = 0). Our S_1_ and T_1_ states for ketene are the same as those Xiao e. al.,^37^ with their work characterizing them as π_⊥_ → π_||_* states, where the π_⊥_ orbital is the second and π_||_* is the fifth.

Our focus in this article is the mechanism of ISC on a mixed state. We will take an exploratory approach that is similar to the reaction path idea that is central to chemistry. Our computations are intended to explore the singlet/triplet crossing region of the potential energy surface in a way similar to that in which one may explore the transition region of a thermal reaction. We now elaborate on this idea briefly.

We start from the fact that passing through a singlet/triplet crossing is a relatively rare event (like passing through a transition state^47−51^). In this way we can draw an analogy with the reaction path idea^52^ that is central to mechanistic chemistry.^53^ The reaction path approach is most commonly used with transition states.^53,54^ It can be realized in dynamics (a dynamical reaction path) by starting a trajectory with the initial momentum in the direction of negative curvature.^48,55^ Alternatively, one could start a trajectory at the “reactants”, with an initial momentum in the direction of the transition state. The results presented in this work are an excited state analogue of this technique; we start from an excited singlet minimum (our “reactants”) (labeled S_1_ in both systems) with activation of the vibrational normal mode that corresponds to motion toward the S/T crossing seam (our “transition state”)—i.e. the normal mode approximately parallel to the gradient difference vector at the minimum energy point on the crossing seam (Figure 1). The essential difference, as we shall show subsequently, is that when the trajectory reaches the crossing seam, it does not proceed to “products” but rather results in a mixture of singlet and triplet states, and after reaching a “turning point” proceeds backward across the seam again. This occurs many times with the population of triplet increasing on each passage of the crossing seam. Thus, we believe that our single “reaction path” trajectory yields some mechanistic insight of ISC.

**Figure 1:**
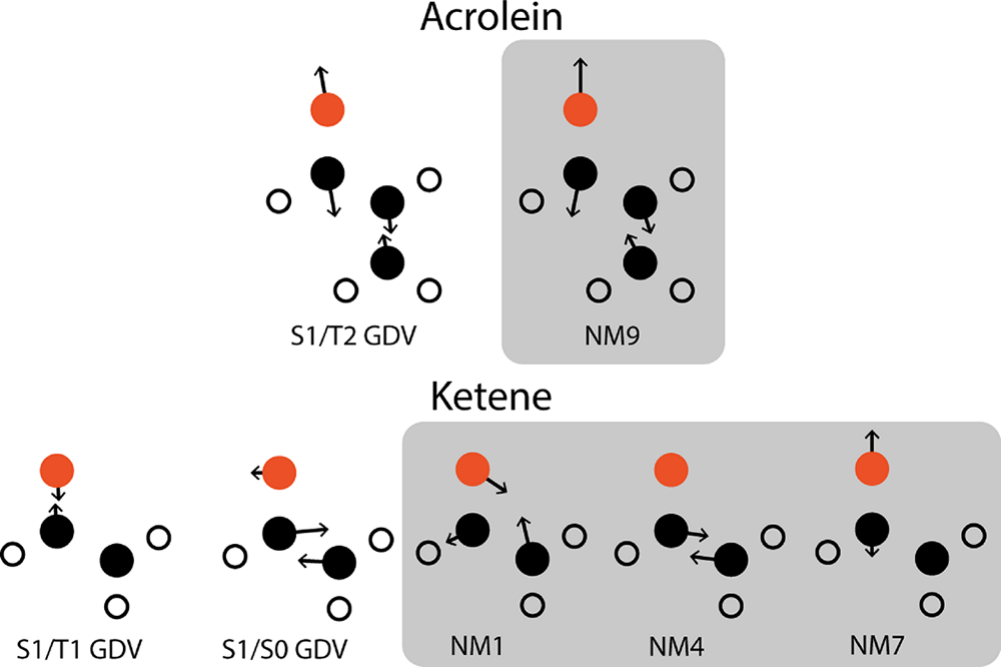
Gradient difference vectors (GDVs) and important normal modes for the dynamics.

In this work we are not attempting to calculate the rate of triplet formation; in our formalism ISC presents itself as a slow buildup of the triplet component of the mixed state.

We now summarize the main chemical concepts associated with ISC as seen in acrolein and ketene. Scheme 2a shows that the acrolein dynamics proceeds with many S_1_/T_2_ recrossings resulting in a continuous increase in the triplet component of the mixed state that develops. For ketene, we have an important effect of the mixing of singlet and triplet on the nuclear motion of the singlet state. We sketch this effect in Scheme 2b,c. Ketene has both a singlet/singlet conical intersection and a triplet/singlet crossing near the starting minima. As we shall show, the mixed S_1_/T_1_ state created by spin–orbit coupling interacts in a different way with S_0_ and thus changes the dynamics of the singlet–singlet nonradiative decay (internal conversion) at the S_0_/S_1_ conical intersection (Scheme 2c). The numerical details will be provided subsequently.

**Scheme 2:**
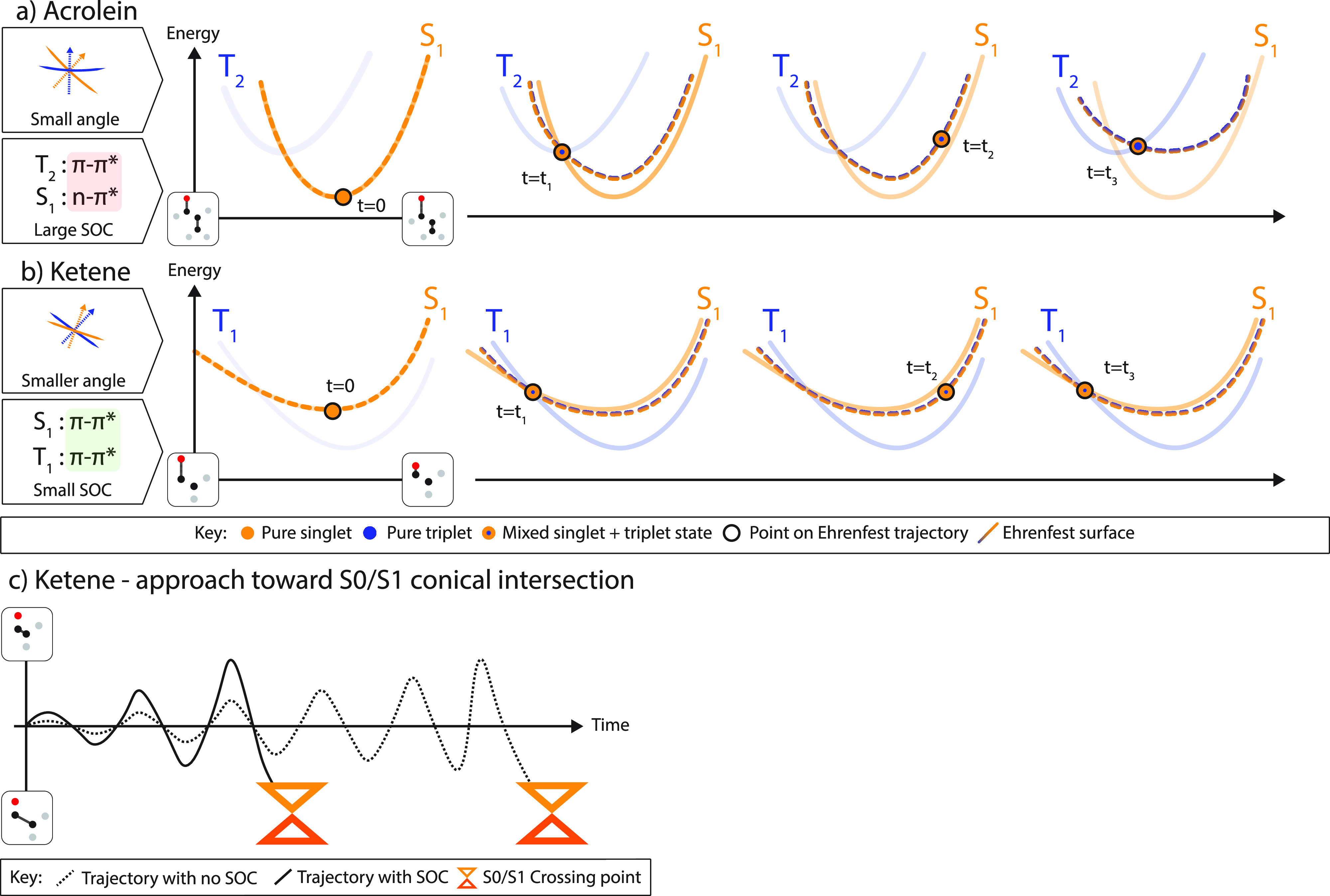
(a, b) Snapshots of the Evolution of the Time-Dependent Ehrenfest Potential Energy Surfaces (Dotted Curve) as a Function of Time (and Parametrically, Mixture of Singlet and Triplet States as Indicated by the Color Shading on the Circles and Curves) in (a) and (b) and (c) Depiction of Including SOC, which Leads to a More Rapid (Time on *x*-Axis) Approach to the Singlet–Singlet Crossing in Ketene by a More Efficient Energy Transfer to the S_0_/S_1_ GDV Normal Mode (*y*-Axis) The adiabatic potential surfaces are shown as solid curves. Notice that the Ehrenfest surface (dotted curve) does not lie on any one adiabatic surface.

We now discuss how we include SOC within the Ehrenfest approximation of the TDSE for electron motion as devised by Vacher et al.^15^

In eq 3*t*_*n*_ is the current timestamp, *t*_*n*–1_ is the previous timestamp, and  is the matrix representation of the CI Hamiltonian in a basis of Slater determinants within a CAS-CI^56^ expansion. We use Slater determinants because we will mix different *S*_*z*_ = 0 states when the SOC is included. The Ehrenfest vector  contains the weights of the Slater determinants in the time-dependent wave function.
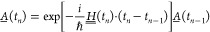
3

The SOC magnitude matrix elements involve only the one-electron operator (eq 2) and are collected in the matrix  in the same basis as  (in fact, it turns out to be convenient to evaluate these matrix elements in the CI eigenvector basis and transform back—the full implementation details are provided in SI), and we recompute this SOC matrix at every time step.
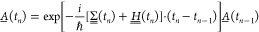
4

Once this electronic propagation step is done, the updated Ehrenfest vector *A* can then be used to compute the molecular properties at each time step—namely the energy, gradient, and Hessian of the electronic wave function.^15^ These quantities are then used to propagate the nuclear wave function. We wish to emphasize that the time-dependent Ehrenfest potential is not a simple average but rather includes the interstate terms in the energy and its derivatives.

Because of the spin–orbit coupling term,  will evolve to become a mixture of singlet and triplet states. Since Slater determinants are not, in general, eigenstates of the spin operator *Ŝ*^2^, to interpret the results, it is necessary to consider a projection of  onto possible states that are either singlet or triplets. The simplest choice is projection of the Ehrenfest vector onto a set of spin pure Hartree–Waller^57^ functions or Clifford Algebra spinors.^58^

The Hartree–Waller (HW) functions^59^ are defined as a singlet
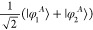
5Or a triplet
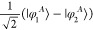
6combination of Slater determinants where |φ_1_^*A*^⟩ and |φ_2_^*A*^⟩ are two determinants that have the same spatial orbitals *A* but with all the spins inverted. Any singlet adiabatic state must be a linear combination of singlet HW functions, and any triplet must be a combination of triplet HW functions.

As previously discussed, upon inclusion of SOC effects, the vector  may have contributions from both triplet and singlet HW functions. If we sum the populations of all pairs of eq 5 and eq 6, we obtain the total triplet and singlet populations of the mixed state.

Finally, if one inspects the individual coefficients of |φ_1_^*A*^⟩ and |φ_2_^*A*^⟩ in  one can distinguish two cases. The coefficients will be equal if the coupling for spatial arrangement A is either a singlet or triplet (this is the situation in acrolein, as we shall show because we mix a *n*–π* singlet with a π–π* triplet). However, if individual coefficients of |φ_1_^*A*^⟩ and |φ_2_^*A*^⟩ in  are not equal then both singlet and triplet states are present with the same spatial arrangement. In this case, the singlet triplet mixed state has the same orbital components *A* (this is the case in ketene where singlet and triplet states are both π–π*).

In acrolein we use a 4SA-CASSCF(6,5)/6-31G* and in ketene a 3SA-CASSCF(6,5)/6-31G* method to generate our starting geometry and orbitals. The relevant states and their dominant configurations are shown in Scheme 1.

In this article both systems (acrolein and ketene) have been studied with the second order Ehrenfest^15^ dynamics method.

The electronic wave function is propagated in a CAS-CI formalism starting from a converged CASSCF calculation (described above) as implemented in a development version of Gaussian DV J05.^60^ This code also computes the gradient and Hessian of the Ehrenfest state. The nuclei are propagated on the Ehrenfest surface using a fifth order predictor corrector scheme of Schlegel^61^ et al. (also, as implemented in Gaussian). For both systems we have used an integrator step size of 10^–2^ (leading to an average time step of ∼0.06 fs). We started the nuclear dynamics (a single representative trajectory) with an initial energy of 20 kcal/mol in acrolein and 24 kcal/mol in ketene in their respective singlet–triplet gradient difference vector (GDV) normal modes. The initial energy in those modes was chosen after some experimentation so that the crossing region was adequately explored because the “reaction path” is somewhat curved.

We shall present the data for 2 simulations for each system with the same initial conditions but for the inclusion of spin–orbit coupling effects. The differences in the resultant nuclear and electronic trajectories illustrate the mechanistic effects of the SOC.

We will now provide the numerical details that support the qualitative picture outlined in Scheme 2.

In both acrolein and ketene, the dynamics is started on the S_1_ state from the S_1_ minimum with an initial momentum of 20 kcal/mol in S_1_/T_2_ GDV and 24 kcal/mol in S_1_/T_1_ GDV, respectively (Scheme 2). From there the trajectory rapidly approaches the singlet/triplet crossing. What happens next depends on the shape of the crossing; in acrolein the S_1_/T_2_ crossing is slightly peaked, and we see that one requires many recrossings of the seam (discussed in Figure 2aii) to reach a significant population of the triplet. In contrast, in ketene, the S_1_/T_1_ crossing is sloped, and the surfaces are almost parallel since the orbital components of the two states are the same (Scheme 1) and the triplet population remains very small (to be discussed in Figure 2bi). This is consistent with the textbook^7,8^ description of ISC as a slow process requiring many recrossings of the singlet–triplet seam (Figure 2aii+bii), from which one would expect a slow transfer of population (Figure 2ai+bi) onto the triplet at every passage of the crossing seam.

**Figure 2:**
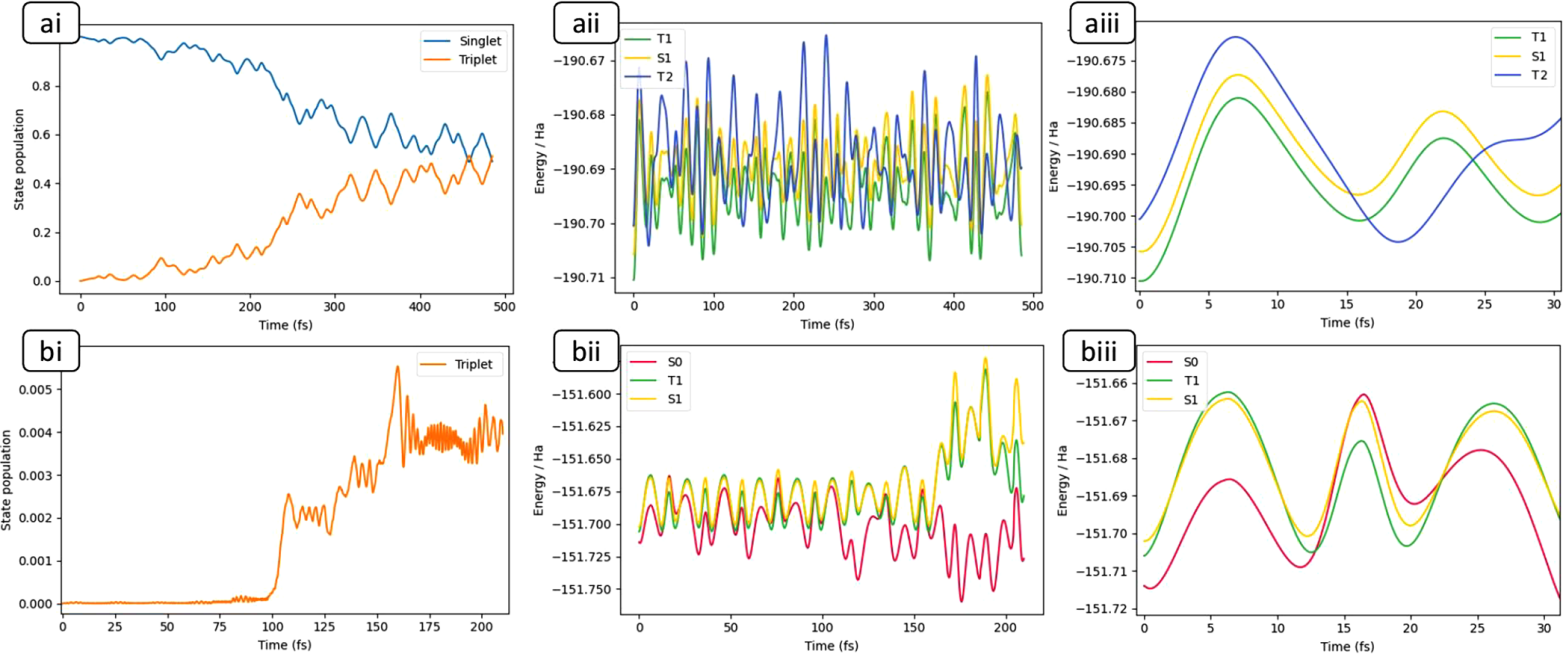
Ehrenfest dynamics for acrolein (a) and ketene (b) starting at their respective S_1_ minima with 20 and 24 kcal/mol in the singlet/triplet GDV normal mode, respectively (Figure 1). *ai + bi*: Population of combined sum of all triplet (and singlet for acrolein) HW components (note the difference in time scale (*x*-axis) and population (*y*-axis)). *aii + bii*: Evolution of time-dependent adiabatic state energies. In ketene, T_1_ and S_1_ are initially parallel and cross with T_2_ as shown in Scheme 2a. *aiii + biii*: Same as aii and bii but zoomed in, to highlight the first few oscillations through S/T crossings and their topography (sloped vs peaked).

For acrolein, as the molecule passes through the S_1_/T_2_ seam (Figure 2aii,aiii), singlet population is transferred onto the triplet (Figure 2ai). As a result, propagation takes place on an effective surface governed by the evolving superposition of the singlet and triplet states (Scheme 2). In the simulation without SOC effects, there is no triplet population as the molecule simply rolls around the S_1_ minima well. The associated effect of S_1_/T_2_ mixing on geometry changes is discussed in the SI (Figure S2). There we show a C–C–C bending normal mode (SI Figure S2) becomes enhanced at around 200 fs, consistent with the sharp rise in triplet population in Figure 2ai and the difference in primary orbital occupancy (S_1_ vs T_2_). Internal conversion from T_2_ to T_1_ is also possible and could be captured by our dynamics but is not observed in our results on the time scale we have simulated.

In ketene, the nuclear dynamics leads to a singlet–singlet conical intersection with a nearby singlet/triplet seam (a 2 singlet +1 triplet system has previously been documented by Granucci et al.^62^). To reach the S_0_/S_1_ seam, one must excite the associated S_0_/S_1_ GDV normal mode, a mixture of NM1 and NM4 (Figure 1). The amplitude of these modes is enhanced with SOC included, similar to acrolein, and thus, the dynamics of the passage across the S_0_/S_1_ conical intersection is changed by spin–orbit coupling. To quantify this effect, we show the paths traced out by the trajectory in normal mode space for the first 150 fs (Figure 3a,b). These plots allow us to explore the correlation of 2 important coupling normal modes of the trajectory, as was done previously^63^ by Lasorne et al. Since there is a transfer of kinetic energy between the *x*-axis normal mode (NM7 primary component of T_1_/S_1_ GDV) and the *y* component (NM1 and NM4 components of S_0_/S_1_ GDV), we observe a Lissajous figure-like trace. These plots show how we reach the singlet/singlet seam—via a slow transfer of energy to the necessary singlet/singlet GDV normal mode.

**Figure 3:**
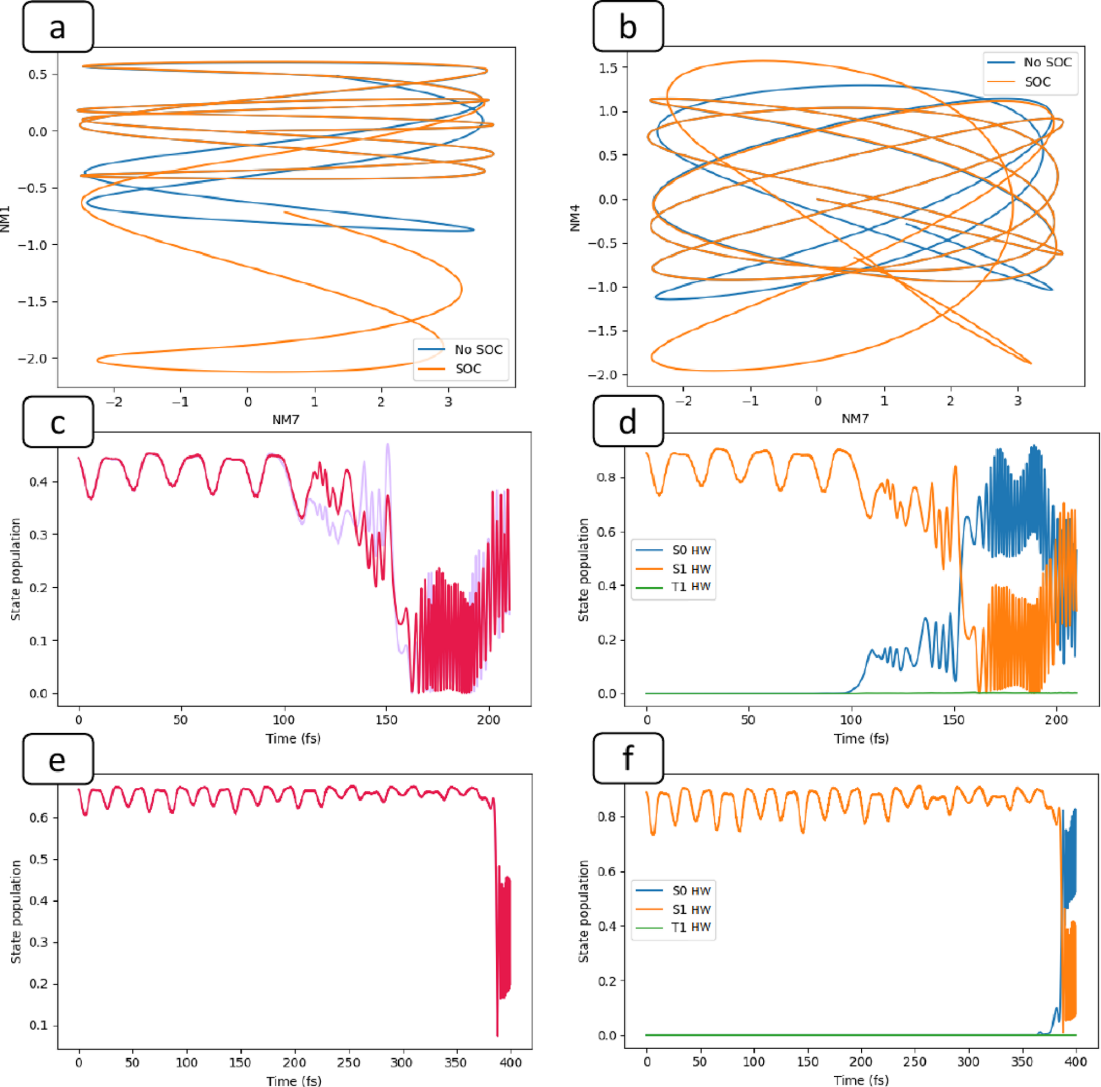
Ketene trajectories with 24 kcal/mol in the S_1_/T_1_ GDV mode. *ab:* Geometry evolution (Lissajous-like figure), plotting NM7 (S_1_/T_1_ GDV; see Figure 1) versus NM4 or NM1 (components of S_1_/S_0_ GDV). Both trajectories start at the S_1_ minima at (0,0). The blue curve is with the spin–orbit interaction disabled, and the orange curve is with the SOC included. The trajectories are truncated at 150 fs for clarity. *c:* The population of 2 dominant Slater determinants with π–π* orbital configuration (|φ_1_^*A*^⟩ and |φ_2_^*A*^⟩ in eqs 5 and 6). Note the splitting after ∼110 fs demonstrating that we are on a mixed singlet/triplet state (due to the phase difference in HW functions). *d:* Population of the dominant HW functions, S_0_HW, etc. (corresponding to Scheme 1). *e, f:* Same as c and d, but for trajectory with SOC disabled.

As a reference point, we shall address the dynamics without a SOC first. As shown in Figure 3a,b,e,f the trajectory slowly takes the system to the S_1_/S_0_ seam after ∼370 fs (Figure 3f).

In Figure 3a–d we show the effect of including SOC for ketene; despite a small triplet population (Figure 2bi) it acts to enhance the transfer of kinetic energy from NM7 to NM1 (Figure 3a,b) and NM4 leading to a faster approach to the S_0_/S_1_ seam (within ∼120 fs, Figure 3d). This transfer of kinetic energy between normal modes is reflected in Figure 3a,b (orange): the trajectory curves with inclusion of SOC gain more *y*-axis amplitude than the ones with SOC effects disabled over the same time frame. The small admixture of T_1_ has thus enhanced the S_1_/S_0_ internal conversion by placing more momentum in the S_0_/S_1_ GDV modes. Notice the rapid oscillation of the diabatic dominant singlet HW populations (Scheme 1) in Figure 3d,f after passing through the conical intersection. This is characteristic of electron dynamics (charge migration).^64^ In fact, the oscillations in the electronic state give rise to the oscillations in the nuclear motion, and the motion of the nuclei induces the electronic dynamics.

For ketene, in particular, we can visualize the singlet–triplet electron dynamics by plotting the two components of the dominant Hartree–Waller function separately [|φ_1_^*A*^⟩ and |φ_2_^*A*^⟩ in eqs 5 + 6]. Without SOC (Figure 3e) the 2 populations remain equal, whereas with SOC on (Figure 3c) the 2 populations diverge due to the phase difference between singlet and triplet HW functions, which clearly illustrates that our dynamics is running on a mixed state.

We have described the mechanism of intersystem crossing as a superposition of singlets and triplets created under the influence of spin–orbit coupling. In this description, the composition of the mixed state controls the nuclear dynamics, rather than the textbook^7,8^ description where one propagates on either a pure singlet or pure triplet surface after the ISC event. Nevertheless, the qualitative ideas about *state populations* involved with intersystem crossing do not change: the coupling of the singlet and the triplet is controlled by passing through the seam of the singlet/triplet conical section and many recrossings are necessary for even partial population transfer. The distinguishing *mechanistic* feature of this work is that the nuclear trajectory is driven by the unique gradient of the coherent superposition of singlet and triplet, which is distinct from the gradient of either singlet or triplet.

In this work, we have reported results for two chemical examples: acrolein with a relatively large spin–orbit coupling and ketene where the spin–orbit coupling is much smaller. In acrolein we saw a large population of triplet, and an effect on geometry (see SI). However, in ketene, the population of triplet was small, but the gradient of the mixed state strongly influenced the singlet/singlet conical intersection approach. El Sayed’s rule suggests that ISC is much faster in acrolein than in ketene, and indeed this agreement is a validation of our approach.

On a point of method, one might expect the surface hop method to yield reasonable results for acrolein but much less so for ketene. We base this prediction on the fact that SOC (and triplet population) is so small in ketene that a majority of trajectories would simply never make the hop onto the triplet—hence never experience the extra excitation of the S_0_/S_1_ GDV normal mode that leads to the faster arrival at the singlet/singlet seam. Of course, one could regard the dynamics presented here as a first step in a hierarchy that might involve many trajectories with sampling, either classical, quantum mechanical,^65,66^ or mixed. However, in these trajectories, if we were to use the Ehrenfest method, we would not need to include a surface hop.

## Supporting Information Available

Additional details on implementation of SOC Ehrenfest algorithm, geometric results for acrolein, starting geometries, and comparison to X2C-CASCI calculation at points of high S/T mixing (PDF)
Transparent Peer Review report available (PDF)

## References

[ref1] Merritt I. C. D.; Jacquemin D.; Vacher M. Attochemistry: Is Controlling Electrons the Future of Photochemistry?. J. Phys. Chem. Lett. 2021, 12 (34), 8404–8415. 10.1021/acs.jpclett.1c02016. 34436903

[ref2] Palacios A.; Martín F. The Quantum Chemistry of Attosecond Molecular Science. WIREs Comput. Mol. Sci. 2020, 10 (1), e1430 10.1002/wcms.1430.

[ref3] Robb M. A.; Jenkins A. J.; Vacher M. How Nuclear Motion Affects Coherent Electron Dynamics in Molecules. In Theoretical and Computational Chemistry Series; Vrakking M. J. J., Lepine F., Eds.; Royal Society of Chemistry: Cambridge, 2018; Chapter 8, pp 275–307. 10.1039/9781788012669-00275.

[ref4] Yarkony D. R. Spin-Forbidden Chemistry within the Breit-Pauli Approximation. Int. Rev. Phys. Chem. 1992, 11 (2), 195–242. 10.1080/01442359209353270.

[ref5] Marian C. M. Spin-Orbit Coupling and Intersystem Crossing in Molecules: Spin-Orbit Coupling. WIREs Comput. Mol. Sci. 2012, 2 (2), 187–203. 10.1002/wcms.83.

[ref6] Fedorov D. G.; Koseki S.; Schmidt M. W.; Gordon M. S. Spin-Orbit Coupling in Molecules: Chemistry beyond the Adiabatic Approximation. Int. Rev. Phys. Chem. 2003, 22 (3), 551–592. 10.1080/0144235032000101743.

[ref7] Turro N. J.; Ramamurthy V.; Scaiano J. C. Modern Molecular Photochemistry of Organic Molecules; University Science Books: Sausalito, CA, 2010.

[ref8] Michl J.; Bonačić-Koutecký V. Electronic Aspects of Organic Photochemistry; Wiley: New York, 1990.

[ref9] Robb M. A. In This Molecule There Must Be a Conical Intersection. In Advances in Physical Organic Chemistry; Elsevier: 2014; Vol. 48, pp 189–228. 10.1016/B978-0-12-800256-8.00003-5.

[ref10] Domcke W.; Yarkony D.; Köppel H. Conical Intersections: Theory, Computation and Experiment; World Scientific Publishing Co. Pte. Ltd.: Singapore, 2012.

[ref11] Yarkony D. R. Conical Intersections: Diabolical and Often Misunderstood. Acc. Chem. Res. 1998, 31 (8), 511–518. 10.1021/ar970113w.

[ref12] Robb M. A. Theoretical Chemistry for Electronic Excited States; Theoretical and computational chemistry series; Royal Society of Chemistry: London, 2018.

[ref13] Kuleff A. I.; Breidbach J.; Cederbaum L. S. Multielectron Wave-Packet Propagation: General Theory and Application. J. Chem. Phys. 2005, 123 (4), 044111 10.1063/1.1961341. 16095350

[ref14] Marquetand P.; Richter M.; González-Vázquez J.; Sola I.; González L. Nonadiabatic Ab Initio Molecular Dynamics Including Spin–Orbit Coupling and Laser Fields. Faraday Discuss. 2011, 153, 261 10.1039/c1fd00055a. 22452086

[ref15] Vacher M.; Mendive-Tapia D.; Bearpark M. J.; Robb M. A. The Second-Order Ehrenfest Method. Theor. Chem. Acc. 2014, 133 (7), 1505 10.1007/s00214-014-1505-6.

[ref16] Danilov D.; Tran T.; Bearpark M. J.; Marangos J. P.; Worth G. A.; Robb M. A. How Electronic Superpositions Drive Nuclear Motion Following the Creation of a Localized Hole in the Glycine Radical Cation. J. Chem. Phys. 2022, 156 (24), 244114 10.1063/5.0093780. 35778090

[ref17] Tran T.; Worth G. A.; Robb M. A. Control of Nuclear Dynamics in the Benzene Cation by Electronic Wavepacket Composition. Commun. Chem. 2021, 4 (1), 48 10.1038/s42004-021-00485-3. 36697520 PMC9814899

[ref18] McQuarrie D. A.; Simon J. D. Physical Chemistry: A Molecular Approach; University Science Books: Sausalito, CA, 1997.

[ref19] Ermler W. C.; Ross R. B.; Christiansen P. A. Spin-Orbit Coupling and Other Relativistic Effects in Atoms and Molecules. In Advances in Quantum Chemistry; Elsevier, 1988; Vol. 19, pp 139–182. 10.1016/S0065-3276(08)60615-2.

[ref20] Pauli W. Zur Quantenmechanik des magnetischen Elektrons. Z. Physik 1927, 43 (9–10), 601–623. 10.1007/BF01397326.

[ref21] Koseki S.; Gordon M. S.; Schmidt M. W. Theoretical Investigation of the Dissociation of Bridged Disilyne. Chem. Phys. Lett. 1992, 200 (3), 303–310. 10.1016/0009-2614(92)80015-4.

[ref22] Bearpark M. J.; Handy N. C.; Palmieri P.; Tarroni R. Spin-Orbit Interactions from Self Consistent Field Wavefunctions. Mol. Phys. 1993, 80 (3), 479–502. 10.1080/00268979300102411.

[ref23] Han B.; Zheng Y. Nonadiabatic Quantum Dynamics in O(3P)+H2→OH+H: A Revisited Study. J. Comput. Chem. 2011, 32 (16), 3520–3525. 10.1002/jcc.21940. 21935969

[ref24] Fu B.; Shepler B. C.; Bowman J. M. Three-State Trajectory Surface Hopping Studies of the Photodissociation Dynamics of Formaldehyde on Ab Initio Potential Energy Surfaces. J. Am. Chem. Soc. 2011, 133 (20), 7957–7968. 10.1021/ja201559r. 21526775

[ref25] Li B.; Han K.-L. Mixed Quantum-Classical Study of Nonadiabatic Dynamics in the O(^3^ P _2,1,0_, ^1^ D _2_) + H _2_ Reaction. J. Phys. Chem. A 2009, 113 (38), 10189–10195. 10.1021/jp904727d. 19722530

[ref26] Cui G.; Thiel W. Generalized Trajectory Surface-Hopping Method for Internal Conversion and Intersystem Crossing. J. Chem. Phys. 2014, 141 (12), 124101 10.1063/1.4894849. 25273406

[ref27] Jenkins A. J.; Liu H.; Kasper J. M.; Frisch M. J.; Li X. Variational Relativistic Two-Component Complete-Active-Space Self-Consistent Field Method. J. Chem. Theory Comput. 2019, 15 (5), 2974–2982. 10.1021/acs.jctc.9b00011. 31018093

[ref28] Klein S.; Bearpark M. J.; Smith B. R.; Robb M. A.; Olivucci M.; Bernardi F. Mixed State ′on the Fly’ Non-Adiabatic Dynamics: The Role of the Conical Intersection Topology. Chem. Phys. Lett. 1998, 292 (3), 259–266. 10.1016/S0009-2614(98)00681-2.

[ref29] Tully J. C. Ehrenfest Dynamics with Quantum Mechanical Nuclei. Chem. Phys. Lett. 2023, 816, 140396 10.1016/j.cplett.2023.140396.

[ref30] Kirrander A.; Vacher M. Ehrenfest Methods for Electron and Nuclear Dynamics. In Quantum Chemistry and Dynamics of Excited States; González L., Lindh R., Eds.; John Wiley & Sons Ltd.: Chichester, U.K., 2020; pp 469–497. 10.1002/9781119417774.ch15.

[ref31] Vacher M.; Bearpark M. J.; Robb M. A. Direct Methods for Non-Adiabatic Dynamics: Connecting the Single-Set Variational Multi-Configuration Gaussian (VMCG) and Ehrenfest Perspectives. Theor. Chem. Acc. 2016, 135 (8), 187 10.1007/s00214-016-1937-2.

[ref32] Abedi A.; Maitra N. T.; Gross E. K. U. Exact Factorization of the Time-Dependent Electron-Nuclear Wave Function. Phys. Rev. Lett. 2010, 105 (12), 123002 10.1103/PhysRevLett.105.123002. 20867633

[ref33] Agostini F.; Gross E. K. U. Ultrafast Dynamics with the Exact Factorization. Eur. Phys. J. B 2021, 94 (9), 179 10.1140/epjb/s10051-021-00171-2.

[ref34] Talotta F.; Morisset S.; Rougeau N.; Lauvergnat D.; Agostini F. Internal Conversion and Intersystem Crossing with the Exact Factorization. J. Chem. Theory Comput. 2020, 16 (8), 4833–4848. 10.1021/acs.jctc.0c00493. 32633509

[ref35] Reguero M.; Olivucci M.; Bernardi F.; Robb M. A. Excited-State Potential Surface Crossings in Acrolein: A Model for Understanding the Photochemistry and Photophysics of. Alpha.,.Beta.-Enones. J. Am. Chem. Soc. 1994, 116 (5), 2103–2114. 10.1021/ja00084a056.

[ref36] Yamamoto N. Derivative MC-SCF and Its Applications to Photochemistry; King’s College London (University of London): 1996.

[ref37] Xiao H.; Maeda S.; Morokuma K. CASPT2 Study of Photodissociation Pathways of Ketene. J. Phys. Chem. A 2013, 117 (32), 7001–7008. 10.1021/jp312719a. 23373716

[ref38] Yamabe S.; Morokuma K. A Theoretical Study on the Paths of Photodissociation: Ketene to Methylene + Carbon Monoxide. J. Am. Chem. Soc. 1978, 100 (24), 7551–7556. 10.1021/ja00492a019.

[ref39] Muñoz Losa A.; Fdez. Galván I.; Aguilar M. A.; Martín M. E. A CASPT2//CASSCF Study of Vertical and Adiabatic Electron Transitions of Acrolein in Water Solution. J. Phys. Chem. B 2007, 111 (33), 9864–9870. 10.1021/jp071993r. 17665941

[ref40] Fang W.-H. A CASSCF Study on Photodissociation of Acrolein in the Gas Phase. J. Am. Chem. Soc. 1999, 121 (36), 8376–8384. 10.1021/ja982334i.

[ref41] Aquilante F.; Barone V.; Roos B. O. A Theoretical Investigation of Valence and Rydberg Electronic States of Acrolein. J. Chem. Phys. 2003, 119 (23), 12323–12334. 10.1063/1.1625363.

[ref42] Bokareva O. S.; Bataev V. A.; Pupyshev V. I.; Godunov I. A. Structure and Dynamics of Acrolein in 1,3(π,Π*) Excited Electronic States: A Quantum-Chemical Study. Spectrochimica Acta Part A: Molecular and Biomolecular Spectroscopy 2009, 73 (4), 654–662. 10.1016/j.saa.2009.03.008. 19419904

[ref43] Hlavacek N. C.; McAnally M. O.; Drucker S. Lowest Triplet (Nπ*) Electronic State of Acrolein: Determination of Structural Parameters by Cavity Ringdown Spectroscopy and Quantum-Chemical Methods. J. Chem. Phys. 2013, 138 (6), 064303 10.1063/1.4789793. 23425467

[ref44] Venturini A.; González J. A CASPT2 and CASSCF Approach to the Cycloaddition of Ketene and Imine: A New Mechanistic Scheme of the Staudinger Reaction. J. Org. Chem. 2002, 67 (25), 9089–9092. 10.1021/jo026188h. 12467436

[ref45] Cui Q.; Morokuma K. *Ab Initio* Study of Nonadiabatic Interactions in the Photodissociation of Ketene. J. Chem. Phys. 1997, 107 (13), 4951–4959. 10.1063/1.474890.

[ref46] Braslavsky S. E. Glossary of Terms Used in Photochemistry, 3rd Edition (IUPAC Recommendations 2006. Pure Appl. Chem. 2007, 79 (3), 293–465. 10.1351/pac200779030293.

[ref47] Miller W. H.; Handy N. C.; Adams J. E. Reaction Path Hamiltonian for Polyatomic Molecules. J. Chem. Phys. 1980, 72 (1), 99–112. 10.1063/1.438959.

[ref48] Bolhuis P. G.; Chandler D.; Dellago C.; Geissler P. L. Transition Path Sampling: Throwing Ropes Over Rough Mountain Passes, in the Dark. Annu. Rev. Phys. Chem. 2002, 53 (1), 291–318. 10.1146/annurev.physchem.53.082301.113146. 11972010

[ref49] Passerone D.; Parrinello M. Action-Derived Molecular Dynamics in the Study of Rare Events. Phys. Rev. Lett. 2001, 87 (10), 108302 10.1103/PhysRevLett.87.108302. 11531506

[ref50] Valsson O.; Tiwary P.; Parrinello M. Enhancing Important Fluctuations: Rare Events and Metadynamics from a Conceptual Viewpoint. Annu. Rev. Phys. Chem. 2016, 67 (1), 159–184. 10.1146/annurev-physchem-040215-112229. 26980304

[ref51] Passerone D.; Ceccarelli M.; Parrinello M. A Concerted Variational Strategy for Investigating Rare Events. J. Chem. Phys. 2003, 118 (5), 2025–2032. 10.1063/1.1533783.

[ref52] Fukui K. The Path of Chemical Reactions - the IRC Approach. Acc. Chem. Res. 1981, 14 (12), 363–368. 10.1021/ar00072a001.

[ref53] Hratchian H. P.; Schlegel H. B. Finding Minima, Transition States, and Following Reaction Pathways on Ab Initio Potential Energy Surfaces. In Theory and applications of computational chemistry; Elsevier: 2005; pp 195–249.

[ref54] Truhlar D. G.; Garrett B. C.; Klippenstein S. J. Current Status of Transition-State Theory. J. Phys. Chem. 1996, 100 (31), 12771–12800. 10.1021/jp953748q.

[ref55] Jung H.; Okazaki K.; Hummer G. Transition Path Sampling of Rare Events by Shooting from the Top. J. Chem. Phys. 2017, 147 (15), 152716 10.1063/1.4997378. 29055335

[ref56] Olsen J.; Roos B. O.; Jo/rgensen P.; Jensen H. J. Aa. Determinant Based Configuration Interaction Algorithms for Complete and Restricted Configuration Interaction Spaces. J. Chem. Phys. 1988, 89 (4), 2185–2192. 10.1063/1.455063.

[ref57] Knowles P. J.; Handy N. C. A New Determinant-Based Full Configuration Interaction Method. Chem. Phys. Lett. 1984, 111 (4–5), 315–321. 10.1016/0009-2614(84)85513-X.

[ref58] Paldus J.; Sarma C. R. Clifford Algebra Unitary Group Approach to Many-electron Correlation Problem. J. Chem. Phys. 1985, 83 (10), 5135–5152. 10.1063/1.449726.

[ref59] Waller I.; Hartree D. On the Intensity of Total Scattering of X-Rays. Proc. R. Soc. London A 1929, 124 (793), 119–142. 10.1098/rspa.1929.0101.

[ref60] Frisch M. J.; Trucks G. W.; Schlegel H. B.; Scuseria G. E.; Robb M. A.; Cheeseman J. R.; Scalmani G.; Barone V.; Petersson G. A.; Nakatsuji H.; Li X.; Caricato M.; Marenich A. V.; Bloino J.; Janesko B. G.; Gomperts R.; Mennucci B.; Hratchian H. P.; Ortiz J. V.; Izmaylov A. F.; Sonnenberg J. L.; Williams; Ding F.; Lipparini F.; Egidi F.; Goings J.; Peng B.; Petrone A.; Henderson T.; Ranasinghe D.; Zakrzewski V. G.; Gao J.; Rega N.; Zheng G.; Liang W.; Hada M.; Ehara M.; Toyota K.; Fukuda R.; Hasegawa J.; Ishida M.; Nakajima T.; Honda Y.; Kitao O.; Nakai H.; Vreven T.; Throssell K.; Montgomery J. A. Jr.; Peralta J. E.; Ogliaro F.; Bearpark M. J.; Heyd J. J.; Brothers E. N.; Kudin K. N.; Staroverov V. N.; Keith T. A.; Kobayashi R.; Normand J.; Raghavachari K.; Rendell A. P.; Burant J. C.; Iyengar S. S.; Tomasi J.; Cossi M.; Millam J. M.; Klene M.; Adamo C.; Cammi R.; Ochterski J. W.; Martin R. L.; Morokuma K.; Farkas O.; Foresman J. B.; Fox D. J. Gaussian, Development Version Rev. J05, Gaussian: 2019.

[ref61] Millam J. M.; Bakken V.; Chen W.; Hase W. L.; Schlegel H. B. *Ab Initio* Classical Trajectories on the Born–Oppenheimer Surface: Hessian-Based Integrators Using Fifth-Order Polynomial and Rational Function Fits. J. Chem. Phys. 1999, 111 (9), 3800–3805. 10.1063/1.480037.

[ref62] Granucci G.; Persico M.; Spighi G. Surface Hopping Trajectory Simulations with Spin-Orbit and Dynamical Couplings. J. Chem. Phys. 2012, 137 (22), 22A501 10.1063/1.4707737. 23249038

[ref63] Lasorne B.; Bearpark M. J.; Robb M. A.; Worth G. A. Controlling S _1_ /S _0_ Decay and the Balance between Photochemistry and Photostability in Benzene: A Direct Quantum Dynamics Study. J. Phys. Chem. A 2008, 112 (50), 13017–13027. 10.1021/jp803740a. 18826292

[ref64] Cederbaum L. S.; Zobeley J. Ultrafast Charge Migration by Electron Correlation. Chem. Phys. Lett. 1999, 307 (3–4), 205–210. 10.1016/S0009-2614(99)00508-4.

[ref65] Jenkins A. J.; Spinlove K. E.; Vacher M.; Worth G. A.; Robb M. A. The Ehrenfest Method with Fully Quantum Nuclear Motion (Qu-Eh): Application to Charge Migration in Radical Cations. J. Chem. Phys. 2018, 149 (9), 094108 10.1063/1.5038428. 30195291

[ref66] Hu W.; Lendvay G.; Maiti B.; Schatz G. C. Trajectory Surface Hopping Study of the O(3P) + Ethylene Reaction Dynamics. J. Phys. Chem. A 2008, 112 (10), 2093–2103. 10.1021/jp076716z. 18088105

