# Supplementary material for: Coherent Mixing of Singlet and Triplet States in Acrolein and Ketene: A Computational Strategy for Simulating the Electron–Nuclear Dynamics of Intersystem Crossing
Source: J Phys Chem Lett. 2023 Jun 26;14(26):6127–34. doi: 10.1021/acs.jpclett.3c01187 (PMC10331830; doi:10.1021/acs.jpclett.3c01187)

Supplementary Information for

Coherent Mixing of Singlet and Triplet States in Acrolein and Ketene: a Computational Strategy for Simulating the Electron-Nuclear Dynamics of Intersystem Crossing

Don Danilov[2a], Andrew J Jenkins[3b], Michael J Bearpark[2c], Graham A Worth[1d], and Michael A Robb[2e]

[1] *Department of Chemistry, University College London, 20 Gordon St., WC1H 0AJ London, United Kingdom*

[2] *Department of Chemistry, Imperial College London, Molecular Sciences Research Hub, 82 Wood Lane, W12 0BZ, London, United Kingdom*

[3] *Department of Chemistry, University of Washington, Seattle, Washington 98195, United States*

Contents

Implementation S1

Ehrenfest vector projection S4

Acrolein: results with no SOC........ S5

Acrolein: Change in nuclear motion with and without SOC S6

Verification of triplet one state approximation with X2C-CASCI computations........ S8

Computed geometries S13

Implementation

The SOC matrix between 2 electronic states $\left|\Psi_j\right\rangle$ and $\left|\Psi_i\right\rangle$ can be defined as follows:

$$\underline{\underline{\Sigma}}_{ij} = \left\langle \Psi_i \middle| \hat{H}_{SO} \middle| \Psi_j \right\rangle$$

We wish to include this effect in a Ehrenfest framework. We start with the basic, non-spin orbit coupled Ehrenfest propagation equation of Vacher et al (The Second-Order Ehrenfest Method - Theor Chem Acc (2014) 133:1505):

$$\underline{A}(t_n) = \underline{\underline{U}}(t_n)\exp\left[-\frac{i}{\hbar}\ \underline{\underline{D}}(t_n)(t_n - t_{n-1})\right]\ \underline{\underline{U}}^{\dagger}(t_n)\underline{A}(t_{n-1})$$

The $\underline{\underline{U}}(t_n)$ matrix corresponds to a unitary transformation from CI basis to a basis of Slater determinants.

We will add the spin orbit coupling magnitude matrix (computed at each timestep) to the diagonal electronic Hamiltonian, $\underline{\underline{D}}(t_n)$

$$\underline{\underline{D'}}(t_n) = \underline{\underline{D}}(t_n) + \underline{\underline{\Sigma}}(t_n)$$

To perform the exponentiation, we need to re-diagonalise with a corresponding unitary transform $\underline{\underline{W}}(t_n)$ that transforms CI basis to a spin-adiabatic CI basis:

$$\underline{\underline{D''}}(t_n) = \underline{\underline{W}}(t_n) \cdot \underline{\underline{D'}}(t_n) \cdot \underline{\underline{W}}^\dagger(t_n)$$

The full propagation equation can then be re-written as

$$\underline{A}(t_n) = \underline{\underline{W}}(t_n)\underline{\underline{U}}(t_n)\exp\left[-\frac{i}{\hbar}\ \underline{\underline{D''}}(t_n)(t_n - t_{n-1})\right]\ \underline{\underline{U}}^\dagger(t_n)\underline{\underline{W}}^\dagger(t_n)\ \underline{A}(t_{n-1})$$

To avoid re-diagonalising the full $\underline{\underline{D}}''(t_n)$ matrix we shall proceed in a pairwise manner; including SOC between a pair of CI states denoted $\left|\Psi_p\right\rangle$, $\left|\Psi_q\right\rangle$.

Note that $\hat{H}_{SO}$ is Hermitian and the SOC magnitude values are real. Hence,

$$\left\langle\Psi_p\middle|\hat{H}_{SO}\middle|\Psi_q\right\rangle = \left\langle\Psi_q\middle|\hat{H}_{SO}\middle|\Psi_p\right\rangle$$

We can define an intermediate SOC matrix:

$$\underline{\underline{\Sigma'_{\{p,q\}}}}(t_n) = \begin{bmatrix} 0 & & & & \\ & \ddots & & & \\ & & 0 & \cdots & \left\langle\Psi_p\middle|\hat{H}_{SO}\middle|\Psi_q\right\rangle & & \\ & & \vdots & \ddots & \vdots & & \\ & & \left\langle\Psi_p\middle|\hat{H}_{SO}\middle|\Psi_q\right\rangle & \cdots & 0 & & \\ & & & & & \ddots & \\ & & & & & & 0 \end{bmatrix}$$

We now compute $\underline{\underline{D'_{\{p,q\}}}}(t_n) = \underline{\underline{D}}(t_n) + \underline{\underline{\Sigma'_{\{p,q\}}}}(t_n)$

$$\underline{\underline{D'_{\{p,q\}}}}(t_n) = \begin{bmatrix} E_1 & & & & \\ & \ddots & & & \\ & & E_p & \cdots & \left\langle\Psi_p\middle|\hat{H}_{SO}\middle|\Psi_q\right\rangle & & \\ & & \vdots & \ddots & \vdots & & \\ & & \left\langle\Psi_p\middle|\hat{H}_{SO}\middle|\Psi_q\right\rangle & \cdots & E_q & & \\ & & & & & \ddots & \\ & & & & & & E_n \end{bmatrix}$$

We now generate a Givens rotation to rotate the off-diagonal element into the diagonal (one step of Jacobi algorithm):

$$\underline{\underline{G_{\{p,q\}}}}(t_n) = \begin{bmatrix} 1 & & & & \\ & \ddots & & & \\ & & \cos\theta & \cdots & -\sin\theta & & \\ & & \vdots & \ddots & \vdots & & \\ & & \sin\theta & \cdots & \cos\theta & & \\ & & & & & \ddots & \\ & & & & & & 1 \end{bmatrix}$$

where

$$\theta = \frac{1}{2}\arctan\left[\frac{2\left\langle\Psi_p \mid \hat{H}_{SO} \mid \Psi_q\right\rangle}{E_p - E_q}\right]$$

We can approximate the diagonal form as follows:

$$\underline{\underline{D''_{\{p,q\}}}}(t_n) \approx \begin{bmatrix} E_1 & & & & & & \\ & \ddots & & & & & \\ & & E'_p & \cdots & 0 & & \\ & & \vdots & \ddots & \vdots & & \\ & & 0 & \cdots & E'_q & & \\ & & & & & \ddots & \\ & & & & & & E_n \end{bmatrix}$$

Where

$$E'_p = \cos^2\theta(E_p - 2\left\langle \Psi_p \mid \hat{H}_{SO} \mid \Psi_q \right\rangle \tan\theta + E_q \tan^2\theta)$$

$$E'_q = \cos^2\theta(\tan^2 E_p + 2\left\langle \Psi_p \mid \hat{H}_{SO} \mid \Psi_q \right\rangle \tan\theta + E_q)$$

We can repeat this process over all pairs of states, updating the energies and unitary transforms with each pair. The total propagation, over all pairs of states is then

$$\underline{\underline{U}}(t_n)\prod_{i,j<i}\underline{\underline{G_{\{i,j\}}}}(t_n)\exp\left[-\frac{i}{\hbar}\,\underline{\underline{D}}"_{\{i,j\}\cdots\{n-1,n\}}(t_n)(t_n - t_{n-1})\right]\prod_{i,j<i}\underline{\underline{G^{\dagger}_{\{i,j\}}}}(t_n)\underline{\underline{U}}^{\dagger}(t_n)$$

Where $\underline{\underline{D}}"_{\{i,j\}\cdots\{n-1,n\}}(t_n)$ is the final diagonal spin orbit coupled energy matrix after all the pairs of states have been rotated in.

Ehrenfest vector projection

Dynamics code provides us with a complex Ehrenfest vector at each timestep: $v \in \mathbb{C}^n$

We want to project on an arbitrary vector $w \in \mathbb{R}^n$ - in practice these are HW functions [Eq 5 + Eq 6 in main text] of the form

$w = \frac{1}{\sqrt{2}}\begin{pmatrix} \vdots \\ 1 \\ \vdots \\ 1 \\ \vdots \end{pmatrix}$ or $w = \frac{1}{\sqrt{2}}\begin{pmatrix} \vdots \\ 1 \\ \vdots \\ -1 \\ \vdots \end{pmatrix}$ where all elements are zero except in the position of spin-flip related slater determinants

Projection then simply involves taking the dot product $w \cdot v$. To convert this complex number into a state vector population we take the modulus and square: $|w \cdot v|^2$

These populations can be then summed across all singlet or triplet HW functions

Acrolein: results with no SOC

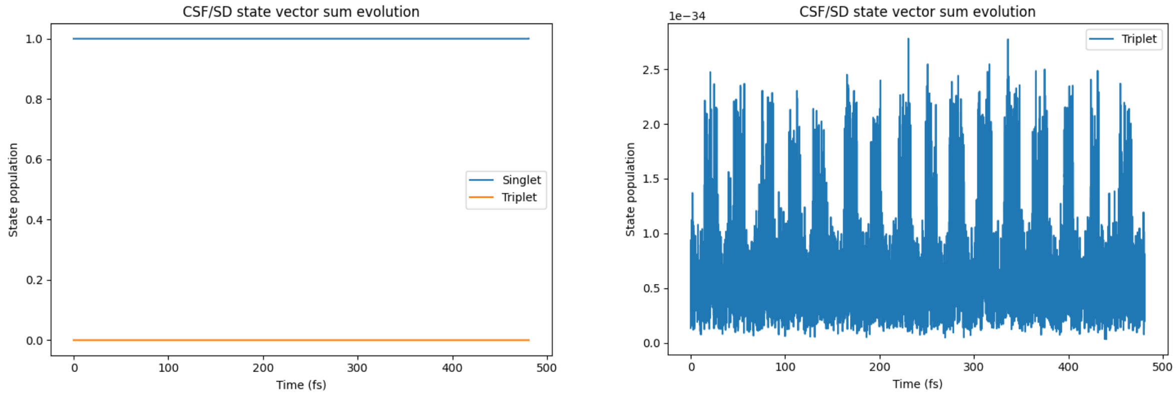

Fig S1 : Populations of the the summed triplet and singlet HW functions (left) and same figure, zoomed in to show triplets only (right)

Observe no triplet population (computes as sum of projections of HW triplet functions) (as should be expected for a job with SOC effects disabled) – minor fluctuation on scale of 10^{-34} is the simply limit of floating-point machine accuracy

Acrolein: Change in nuclear motion with and without SOC

We do observe a geometric difference as compared to trajectory with SOC included, especially in evolution of NM2 and NM8 (reminder – we start both trajectories with 20kcal/mol in NM9):

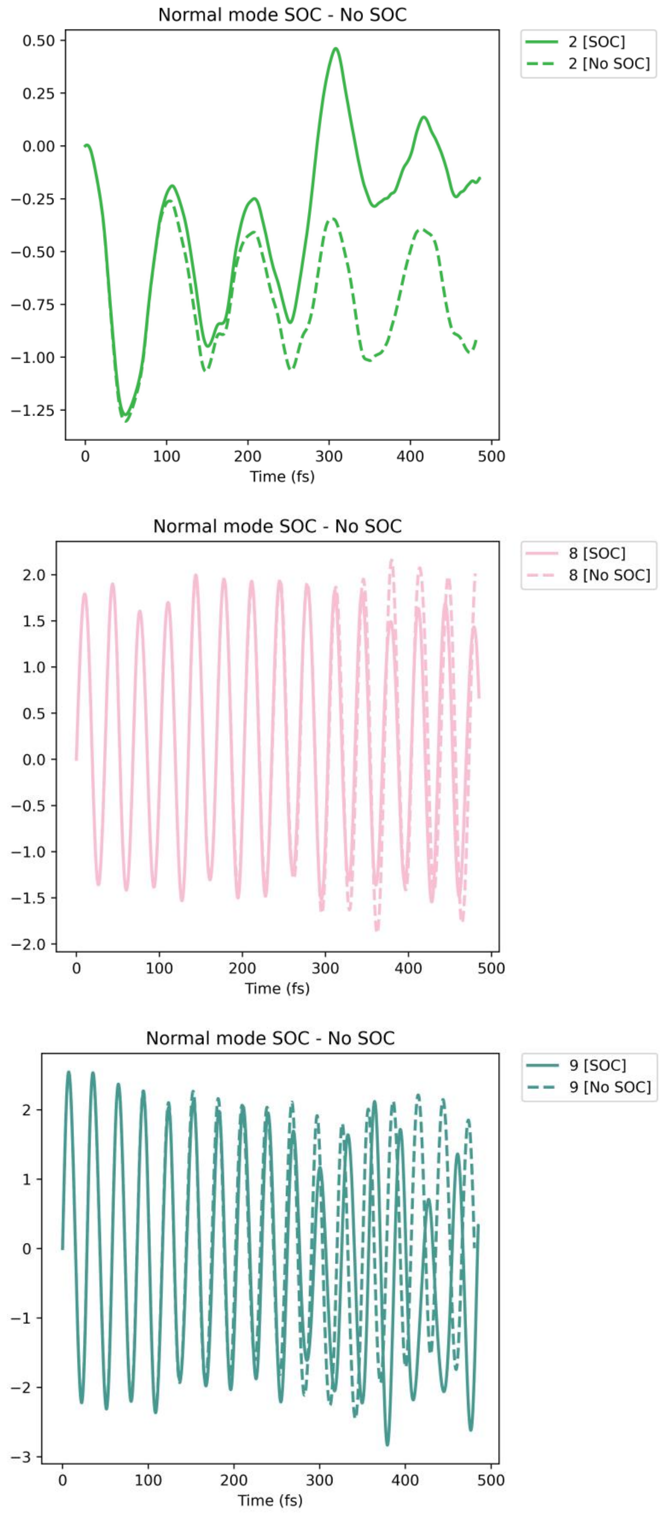

Fig S2: Change in normal mode evolution for NM2 [compound C-C-C bend], NM8 and NM9 with SOC effects included (unbroken) and disabled (dashed).

Note that NM2 and NM8 are compound stretch-bends

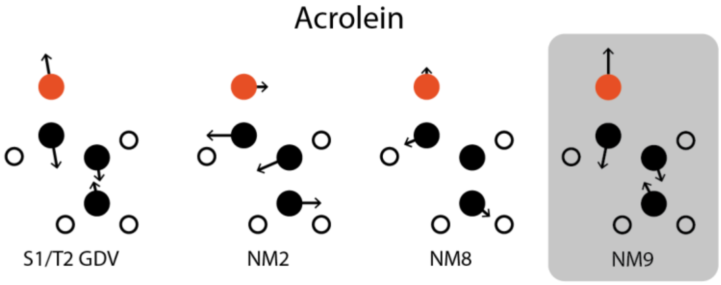

Fig S3 : Schematic representations of the vibrational normal modes

This is wholly consistent with change in primary orbital occupancy involved in S_1 (O lp → π*) → T_2 (π → π*) as discussed in the main text.

Verification of triplet one state approximation with X2C-CASCI computations

To verify the average triplet approximation, we have selected a few points of high singlet/triplet mixing along the trajectories to re-compute with a X2C-CASSCF code - A. J. Jenkins, H. Liu, J. M. Kasper, M. J. Frisch, X. Li, "Variational Relativistic Two-Component Complete-Active-Space Self-Consistent Field Method". *J. Chem. Theory Comput.* (**2019**) *15*, 2974

The results from the X2C-CASCI calculation (avoid re-optimising the orbitals) are shown on the left, the result from our dynamics in the table on the right with SOC values reported between highlighted states. Note the high degree of singlet triplet mixing (indicated by intermediate S values $0 < S < 1$) and the small triplet microstate splitting - on the scale of $\sim 10^{-4}$ Hartree or below. This confirms that our trajectories are indeed visiting geometries where singlet triplet mixing is strong and that our approximation of treating the 3 microstate triplets as one is a reasonable one for these systems.

For example, at Acrolein geometry 246 (15.3fs) on the left the X2C-CASCI results, state 1 is the S_0 (suggested by ordering of the adiabatic states from our dynamics shown in box on the far-right hand side). Next 3 states (2,3,4) are the 3 triplet components corresponding to T_1. These states are split by a tiny amount (2.7E-5 Hartree). Next 3 states (5,6,7) are the T_2 triplets of interest – state 5 shows a high degree of S/T mixing (S value of 0.805 implying a significant S/T mixing) and lastly state 8 is the S_1 singlet of interest – it's very spin-mixed (S=0.393). The actual triplet splitting with T_2 is 2.18E-4 Hartree – this again validates the use of the average triplet approximation.

Acrolein

Geometry 246 - 15.30fs

State	Energy (Hartree)	S	Triplet Splitting
1	-190.8605853	0.000	-
2	-190.7914253	1.000	-
3	-190.7914253	1.000	-
4	-190.7913987	1.000	2.66E-05
5	-190.7878060	0.805	-
6	-190.7875879	1.000	-
7	-190.7875879	1.000	-
8	-190.7870996	0.393	2.18E-04

State	Energy	Spin
1	-190.7697822	S_0
2	-190.7006085	T_1
**3**	**-190.6967806**	$\mathbf{T_2}$
**4**	**-190.6964920**	$\mathbf{S_1}$

SOC -0.0002942419

Geometry 408 - 25.06fs

State	Energy (Hartree)	S	Triplet Splitting
1	-190.8576103	0.000	-
2	-190.7849294	1.000	-
3	-190.7849294	1.000	-
4	-190.7849110	1.000	1.84E-05
5	-190.7810285	0.135	-
6	-190.7799836	1.000	-
7	-190.7799836	1.000	-
8	-190.7799154	0.948	6.82E-05

State	Energy	Spin
1	-190.7666873	S_0
2	-190.6940117	T_1
**3**	**-190.690035**	$\mathbf{T_2}$
**4**	**-190.6890501**	$\mathbf{S_1}$

SOC -0.0002767791

Geometry 737 - 44.40fs

State	Energy (Hartree)	S	Triplet Splitting
1	-190.8593469	0.000	-
2	-190.7841489	1.000	-
3	-190.7841489	1.000	-
4	-190.7841271	1.000	2.17E-05
5	-190.7800698	0.796	-
6	-190.7798601	1.000	-
7	-190.7798601	1.000	-
8	-190.7794036	0.405	2.10E-04

State	Energy	Spin
1	-190.7685807	S_0
2	-190.6933745	T_1
**3**	**-190.6890846**	$\mathbf{T_2}$
**4**	**-190.6888298**	$\mathbf{S_1}$

SOC -0.0002809793

Geometry 833 - 49.91fs

State	Energy (Hartree)	S	Triplet Splitting
1	-190.8500079	0.000	-
2	-190.7834089	1.000	-
3	-190.7834089	1.000	-
4	-190.7833848	1.000	2.41E-05
5	-190.7799577	0.485	-
6	-190.7795339	1.000	-
7	-190.7795339	1.000	-
8	-190.7793312	0.737	2.03E-04

State	Energy	Spin
1	-190.7591532	S_0
2	-190.6925512	T_1
**3**	**-190.6888903**	$\mathbf{S_1}$
**4**	**-190.6886756**	$\mathbf{T_2}$

SOC -0.0002810088

Geometry 1769 - 106.51fs

State	Energy (Hartree)	S	Triplet Splitting
1	-190.8436706	0.000	-
2	-190.7780429	1.000	-
3	-190.7780429	1.000	-
4	-190.7780100	1.000	3.29E-05
5	-190.7752123	0.908	-
6	-190.7750589	1.000	-
7	-190.7750589	1.000	-
8	-190.7743043	0.220	1.53E-04

State	Energy	Spin
1	-190.7528154	S_0
2	-190.687173	T_1
**3**	**-190.6842085**	$\mathbf{T_2}$
**4**	**-190.6835805**	$\mathbf{S_1}$

SOC -0.0002894328

Geometry 1983 - 117.24fs

State	Energy (Hartree)	S	Triplet Splitting
1	-190.8657705	0.000	-
2	-190.7889869	1.000	-
3	-190.7889869	1.000	-
4	-190.7889775	1.000	9.42E-06
5	-190.7837160	0.004	-
6	-190.7768325	1.000	-
7	-190.7768325	1.000	-
8	-190.7768258	0.999	6.72E-06

State	Energy	Spin
1	-190.7749537	S_0
2	-190.6981749	T_1
**3**	**-190.6928861**	$\mathbf{S_1}$
**4**	**-190.6859913**	$\mathbf{T_2}$

SOC -0.0002964590

Table S1 : Comparison of our dynamics results at points of high S/T mixing with X2C-CASCI for acrolein

Ketene

Geometry 499 - 30.48fs

State	Energy (Hartree)	S	Triplet Splitting
1	-151.7884165	0.000	-
2	-151.7675289	1.000	-
4	-151.7675251	1.000	3.83E-06
5	-151.7674040	0.000	-

State	Energy	Spin
1	-151.7116521	S_0
**2**	**-151.6906966**	$\mathbf{T_1}$
**3**	**-151.6905744**	$\mathbf{S_1}$

SOC -0.0000013016

Geometry 1407 - 82.06fs

State	Energy (Hartree)	S	Triplet Splitting
1	-151.7678123	0.004	-
2	-151.7618141	0.003	-
3	-151.7616373	1.000	-
5	-151.7616257	0.998	1.16E-05

State	Energy	Spin
1	-151.6910825	S_0
**2**	**-151.6850302**	$\mathbf{S_1}$
**3**	**-151.6848498**	$\mathbf{T_1}$

SOC -0.0000061195

Geometry 1756 - 102.12fs

State	Energy (Hartree)	S	Triplet Splitting
1	-151.7587807	0.352	-
2	-151.7585201	1.000	-
4	-151.7584742	0.819	4.59E-05
5	-151.7567832	0.032	-

State	Energy	Spin
**1**	**-151.681934**	$\mathbf{S_1}$
**2**	**-151.681727**	$\mathbf{T_1}$
3	-151.6800742	S_0

SOC -0.0001262024

Geometry 1857 - 109.82fs

State	Energy (Hartree)	S	Triplet Splitting
1	-151.7829649	0.001	-
2	-151.7658464	0.985	4.10E-07
3	-151.7658461	1.000	-
5	-151.7657482	0.084	-

State	Energy	Spin
1	-151.7062307	S_0
**2**	**-151.6890455**	$\mathbf{T_1}$
**3**	**-151.6889558**	$\mathbf{S_1}$

SOC -0.0000188567

Geometry 2107 - 121.83fs

State	Energy (Hartree)	S	Triplet Splitting
1	-151.8019447	0.000	-
2	-151.7580487	0.071	-
3	-151.7579829	1.000	-
5	-151.7579780	0.975	4.83E-06

State	Energy	Spin
1	-151.7251709	S_0
**2**	**-151.6812069**	$\mathbf{S_1}$
**3**	**-151.6811419**	$\mathbf{T_1}$

SOC -0.0000125421

Table S2 : Comparison of our dynamics results at points of high S/T mixing with X2C-CASCI for ketene

Computed geometries

Ketene

S1 Minima

C	1.079314	-0.481821	-0.000000
H	2.092748	-0.137530	0.000000
H	0.877379	-1.536621	-0.000000
C	-0.000000	0.458581	0.000000
O	-1.180751	0.226699	0.000000

S1/T1 Crossing

C	1.083697	-0.468039	0.000441
H	2.103645	-0.140210	-0.000671
H	0.849663	-1.512380	-0.000189
C	0.052367	0.447521	0.000670
O	-1.220682	0.202416	-0.000251

Gradient Difference Vector S1-T2

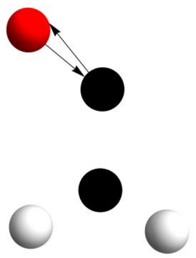

S1/S0 Crossing

C	1.080179	-0.473490	-0.000000
H	2.105483	-0.162585	0.000000
H	0.852225	-1.523030	0.000000
C	-0.008173	0.519582	0.000000
O	-1.161024	0.168831	-0.000000

S0-S1 GDV

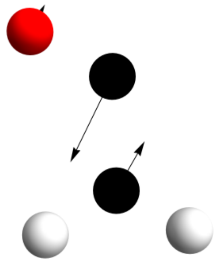

S0-S1 Derivative Coupling (out of plane)

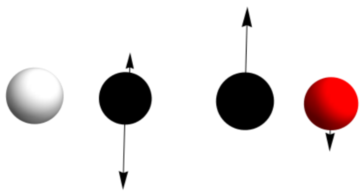

Acrolein

S1 Minima

C	1.201708	1.377256	-0.000000
C	0.000000	0.657209	-0.000000
C	-0.071208	-0.714864	0.000000
O	-1.248062	-1.384177	0.000000
H	2.153921	0.880023	-0.000000
H	1.200208	2.449229	-0.000000
H	-0.928714	1.201951	0.000000
H	0.776085	-1.375395	-0.000000

S1-T2 Crossing

C	-0.120554	-0.719599	0.000000
C	0.005093	0.636744	0.000000
C	1.283899	1.333172	-0.000000
O	-1.316219	-1.307052	-0.000000
H	0.730273	-1.380477	0.000000
H	-0.896413	1.222833	0.000000
H	1.321116	2.403482	-0.000000
H	2.208607	0.789407	-0.000000

S1-T2 GDV

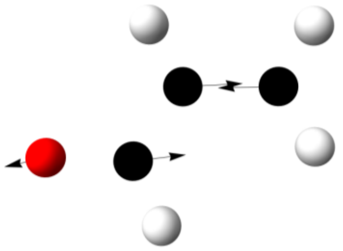

Supplement: Supplementary file 1 — jz3c01187_si_001.pdf [file jz3c01187_si_001.pdf]
