# Supplementary material for: Coherent Mixing of Singlet and Triplet States in Acrolein and Ketene: A Computational Strategy for Simulating the Electron–Nuclear Dynamics of Intersystem Crossing
Source: J Phys Chem Lett. 2023 Jun 26;14(26):6127–34. doi: 10.1021/acs.jpclett.3c01187 (PMC10331830; doi:10.1021/acs.jpclett.3c01187)
Supplement: Supplementary file 2 — jz3c01187_si_002.pdf [file jz3c01187_si_002.pdf]

jz-2023-01187t.R1

Name: Peer Review Information for "Coherent Mixing of Singlet and Triplet States in Acrolein and Ketene: A Computational Strategy for Simulating the Electron-Nuclear Dynamics of Intersystem Crossing"

First Round of Reviewer Comments

Reviewer: 1

Comments to the Author

The two essential objections of this reviewer are as follows.

(1) The propagation of the trajectory on an intermediate (average) potential energy surface is, by construction, an artefact of the Ehrenfest approximation and is not a physical phenomenon. It is well known (see, for example, D. Kohen et al., JCP 109, 4713 (1998) or G. Stock and M. Thoss, Adv. Chem. Phys. 131, 243 (2005)) that the Ehrenfest approximation can be accurate when electronic states are quickly and heavily mixed within a limited range of nuclear coordinate space, as is the case for rapid relaxation through easily accessible conical intersections. Intersystem crossing (ISC) is perhaps the least suitable problem for application of the Ehrenfest approximation. The surface-hopping algorithm is intrinsically better adapted to the physics of ISC. Application of Fermi's Golden rule is the most efficient way of treating ISC for molecules with first-row atoms.

(2) A single quasi-classical trajectory does not carry physically relevant information. In all classical-path methods, a converged sampling of the quantum mechanically defined initial condition is indispensable. In the present example, this could be, e.g., the nuclear Wigner distribution at the energy minimum of the S1 state. Only converged averages of observables, such as electronic state populations, are physically meaningful.

Other minor comments.

(i) The Ehrenfest method is the most time-honoured approximation for quasi-classical nonadiabatic dynamics. It is strange that only self-citations are used when referring to the Ehrenfest method.

(ii) The matrix elements of the Breit-Pauli operator with real-valued basis functions are purely imaginary. It is not explained why a real symmetric spin-orbit matrix is assumed in the SI.

(iii) There are strange inaccuracies in the text. The step size is reported in coordinate space rather than in time, as appropriate for the solution of the time-dependent Schroedinger equation. The initial momentum is given in kcal/mol (??).

(iv) The rapid oscillations after internal conversion to the S0 state are unrelated to electron dynamics. They reflect the large vibrational excess energy in the S0 state after internal conversion.

(v) The conclusion that ISC is much faster in acrolein than in ketene is rather trivial. This follows from the El Sayed rules without any dynamics simulations.

This work does not provide new physical insights and cannot be recommended for publication.

Reviewer: 2

Comments to the Author

The manuscript "Coherent mixing of singlet and triplet states in acrolein and ketene: A computational strategy for simulating the electron-nuclear dynamics of intersystem crossing (ISC)" by Danilov et al. studies the effect of spin-orbit coupling on the excited-state dynamics in acrolein and ketene. The main message of this work is how the coherent mixing of singlet and triplet states affects the nuclear dynamics, via the average time-dependent Ehrenfest potential, and, in turn, the slow population transfer from the initially populated singlet to the triplet. In particular, in ketene, the spin-orbit coupling is very small compared to acrolein (at the S1 minimum where the trajectories are initialized). Nonetheless, the authors observe a strong effect on the dynamics of ketene, since the behavior of the system at the S0/S1 conical intersection is different when simulations w/o spin-orbit coupling are compared.

I find the manuscript well written and the message quite clear. I would recommend it for publication in The Journal of Physical Chemistry Letters, even though I have some general concerns with the employed methodology itself.

It seems to me that the authors make quite strongly the point that it is the Ehrenfest method, along with its average potential driving the nuclear dynamics, that yields the observed effects (for instance, modification of the S0/S1 dynamics in ketene upon inclusion of spin-orbit coupling). Surface hopping might give different results because the nuclear dynamics is propagated either on singlet potentials or on triplet potentials. Therefore, what is correct? I am not so sure that the average potential is the correct answer. For attosecond dynamics, Ehrenfest might be a good approximation. However, for long-time dynamics, I would expect decoherence effects to be important and, thus, a surface hopping treatment, with proper decoherence corrections, might yield results closer to the "chemical reality" (see, for instance, J. Chem. Phys. 154 (2021) 094310 by the Truhlar group). I would like to ask the authors (1) if they could comment on the different dynamics that they would expect with a surface-hopping-like approach (AIMS would probably be similar and the exact factorization would yield something in between), and (2) why they think that their Ehrenfest dynamics describes better the reality of acrolein and ketene dynamics.

Another point that I would like the authors to comment on is the following. They report that the spin-orbit coupling at the S1 minimum, where the trajectory is initialized, in ketene is much smaller than acrolein. What about along the geometries visited by the trajectories? Do the authors use a constant value of the spin-orbit coupling? Is it possible to know if this is a good assumption by computing the spin-orbit coupling at some additional geometries?

Reviewer: 3

Comments to the Author

In this letter, the authors address a very interesting issue, namely the question whether spin–orbit mixed Born-Oppenheimer states are favorably used as starting points for a nuclear dynamics simulation of intersystem crossing processes. To this end, they set up an Ehrenfest dynamics scheme within the CAS-CI formalism, using Slater determinants with spin magnetic quantum number of MS=0 that can represent singlet as well as triplet states. Multiplicity mixing is achieved by inclusion of an empirical one-electron spin–orbit operator.

The performance of the method is examined for two example molecules, i.e., acrolein and ketene, for which they carry out dynamics simulations with and without spin–orbit coupling

and compare the results. The theoretical approach appears very promising for handling fast intersystem crossing processes, but there are still some open questions. Moreover, the reviewer has significant difficulties with the application part of the letter.

1) In the supporting information (page S2), the authors state that Hso is Hermitian and the SOC values are real. While the first statement is true, indeed, the second is not in general. Matrix elements of Hso typically are complex-valued. Can the authors describe in more detail why the SOC values are supposed to be real in their particular examples?

2) In molecules with small SOC values, huge non-adiabatic couplings between the spin–orbit coupled states are expected to arise in the neighborhood of a singlet–triplet crossing. How are these handled in the proposed approach?

3) In the acrolein case, the authors examine the intersystem crossing between the S1 and T2 states which is an El-Sayed allowed process. They interpret their results in such a way that many re-crossings of the seam are required to reach a significant population of the triplet. Can they deduce a rate constant from their simulations? And how realistic is a multiple crossing back and forth between T2 and S1 given that the internal conversion from T2 to T1 presumably is competitive. Can the authors include this decay process in their simulations to get a more realistic picture?

4) There is a severe problem with the ketene case. In the text and in Scheme 2b), the authors designate its S1 and T1 states as π-π* states, but their dominant configurations in Scheme 1 rather point towards a π-σ* excitation. The occupation of the σ* orbital appears to be the reason why the nuclear dynamics lead to a S1-S0 conical intersection. However, neither π-π* nor π-σ* characteristics represent the electronic structures of the S1 and T1 states correctly. According to Szalay et al. (P. G. Szalay; A. G. Császár; L. Nemes. J. Chem. Phys. 105, 1034 (1996) https://doi.org/10.1063/1.471948 and references therein), the S1 and T1 states of ketene rather originate from an n-π* excitation. This gives rise to the question whether the ketene results presented in this letter are meaningful at all. Moreover, the potential energy surfaces of a 3π-π* and 1π-π* pair of states with equal spatial orbital occupation ought to be separated by a substantial energy gap. How can it happen that their potential energy surfaces cross as suggested in Scheme 2b?

5) Scheme 1: Why do the authors indicate a spin magnetic quantum number of MS=1 for the triplet states although they employ the MS=0 component in their calculations?

6) Please define TDSE.

Author's Response to Peer Review Comments:

We have attached the summary of editorial changes and the response to referees

Reply to referees

NOTE: Referee comments in red, Our reply in black, Changes to paper and SI in blue

The changes in the manuscript are marked in red, in the marked-up copy

Reviewer: 1

The two essential objections of this reviewer are as follows.

(1) The propagation of the trajectory on an intermediate (average) potential energy surface is, by construction, an artefact of the Ehrenfest approximation and is not a physical phenomenon. It is well known (see, for example, D. Kohen et al., JCP 109, 4713 (1998) or G. Stock and M. Thoss, Adv. Chem. Phys. 131, 243 (2005)) that the Ehrenfest approximation can be accurate when electronic states are quickly and heavily mixed within a limited range of nuclear coordinate space, as is the case for rapid relaxation through easily accessible conical intersections. Intersystem crossing (ISC) is perhaps the least suitable problem for application of the Ehrenfest approximation. The surface-hopping algorithm is intrinsically better adapted to the physics of ISC.

We would argue that in fact, Ehrenfest is better suited to describe the nuclear geometric effects of ISC than surface hop. We hope that Referee 1 might accept the following rebuttal.

The surface hop method describes the transition from Singlet -> Triplet in a binary way. The gradient following the transition is either that of the singlet or the triplet only. In the approach we used here, the gradient corresponds to a mixed singlet/triplet state. The time-dependant Schrödinger equation for electronic motion is a common feature of both surface hop where it is used to compute hop probability (and the gradient is that of the selected adiabatic state) and Ehrenfest where the gradient is computed directly from the TDSE mixed state. The effect on the geometry in a surface hop approach is only seen by averaging a number of trajectories, whereas in Ehrenfest a single trajectory is seen directly as a result of the mixed state.

Of course, one could regard the dynamics presented here as a first step in a hierarchy that may involve many trajectories, either classical, quantum mechanical or mixed. However, in these trajectories if we were to use the Ehrenfest method we would not need to include a surface hop.

Have added this point to the conclusions [Pg 12]

Application of Fermi's Golden rule is the most efficient way of treating ISC for molecules with first-row atoms.

Fermi's golden rule gives a transition rate constant. We do not attempt to compute a rate in our calculations, rather we are trying to investigate molecular changes occurring in the transition region and immediately afterwards.

The most common formulation of Fermi's Golden rule between discrete quantum states (n & m) with a perturbation W – spin orbit coupling in this case - is $\frac{1}{\tau_{m\to n}} \approx \frac{2\pi}{\hbar}\left|\langle n|W|m\rangle\right|^2 \delta(E_m - E_n)$. For surface hop type dynamics this could be used to describe Singlet/Triplet transitions at points along the seam (where $E_m = E_n$) as has previously been implemented (see for example the work of Hu et al J. Phys. Chem. A 112, 2093-2103 (2008) where the hops only occur only on the S/T seam) together with some probabilistic transfer based on the rate, whereas our method can have small (and critically **incomplete**) population transfers occurring near the crossing region.

In our computations we always end up on a mixed state – thus ISC is a slow build-up of triplet population. Application of Fermi's Golden Rule as described above results in in monotonic population transfer between coupled states - which isn't what we document here.

The paper has been modified – we have now discussed the concept of a rate in the introduction [Pg 3]

(2) A single quasi-classical trajectory does not carry physically relevant information. In all classical-path methods, a converged sampling of the quantum mechanically defined initial condition is indispensable. In the present example, this could be, e.g., the nuclear Wigner distribution at the energy minimum of the S1 state. Only converged averages of observables, such as electronic state populations, are physically meaningful.

We agree in general! But we hope the referee will consider the following discussion where we will try to summarise a general perspective of the technique that we use, and we have placed an abridged version in the main text. In this work we wanted to explore a region of the potential energy surface as opposed to attempting to calculate some experimental observable (such as a rate). A single trajectory related to the reaction path (a dynamical reaction path) concept should be a reasonable technique to achieve this goal.

The approach we use here is closely related to the reaction path idea that is central to mechanistic chemistry (Yang & Houk (2018) 10.1002/chem.201706032 and Hratchian & Schlegel (2005) 10.1016/B978-044451719-7/50053-6 and Miller et al (1980) 10.1063/1.438959).

The reaction path approach (IRC Fukui (1981) 10.1021/ar00072a001) is often used with transition states (Hratchian & Schlegel (2005) 10.1016/B978-044451719-7/50053-6). Starting at the transition state, one starts a steepest decent in mass-weighted coordinates with initial displacement in the direction of negative curvature. However, there is no acceleration in this approach. One could also run a trajectory with the initial momentum in the direction of negative curvature (see for example Jung et al (2017) 10.1063/1.4997378 and Bolhuis et al (2002) 10.1146/annurev.physchem.53.082301.113146). In this work, we are concerned with the reverse problem, namely passage through the transition state (singlet/triplet crossing seam) from a reactant minima. However, passage through a transition state is a rare event (see for example, Passerone et. al (2003) 10.1063/1.1533783). Many techniques are used to avoid this problem (such as flooding – Ray et al (2022) 10.1021/acs.jctc.2c00806). Another technique (which we used in this work) involves running a trajectory backwards from a minima toward the transition state. The approach we have used identifies the singlet triplet conical intersection seam as the transition state and runs a trajectory with initial momentum in the direction (the reverse of the transition vector) pointing towards the crossing seam. The essential difference is that when the trajectory reaches the crossing seam, it does not proceed to 'products' but rather results in a mixture of singlet and triplet states, and after reaching a 'turning point' proceeds backwards across the seam again. This occurs many times with the population of triplet increasing on each passage of the crossing seam. This is the initial step in trying to understand ISC mechanism in terms of state occupations and nuclear geometric effects.

We accept that a possible next step might be to run dynamics starting at the same point with usual sampling. Our objective was to understand the mechanism and mixed state population via recrossing the singlet/triplet seam. We believe that the reaction path concept as we have used it in our Ehrenfest dynamics here provides a new insight into the mechanism of ISC. The feature that is

missing is the coupling of the other trajectories to the sample. But this is ignored by surface hop approach as well except that the momentum in the derivative coupling is added in an ad-hoc way.

We have modified the manuscript to emphasise these objectives [Pg 3]

Other minor comments.

(i) The Ehrenfest method is the most time-honoured approximation for quasi-classical nonadiabatic dynamics. It is strange that only self-citations are used when referring to the Ehrenfest method.

Of course, we have refenced the details of our implementation of Ehrenfest based on our earlier work, which is distinct because of the computation of the intrastate gradient components. We have now added, to provide some background, a number of recent reviews - Tully 2023, Vacher 2016 and Kirrander 2020 to redress this. [Pg 3]

(ii) The matrix elements of the Breit-Pauli operator with real-valued basis functions are purely imaginary. It is not explained why a real symmetric spin-orbit matrix is assumed in the SI.

We use only the SOC magnitude which will always be real.

The text and SI was updated to reflect this [Pg 6 – after Eq 3]

(iii) There are strange inaccuracies in the text. The step size is reported in coordinate space rather than in time, as appropriate for the solution of the time-dependent Schrödinger equation.

We use a Predictor-Corrector integration scheme wherein the step size in time and coordinate space is variable.

We have now clarified this in the text [Pg 8]

The initial momentum is given in kcal/mol (??).

In the text we provide the kinetic energy as opposed to momentum.

We stated this explicitly in the text [Pg 8]

(iv) The rapid oscillations after internal conversion to the S0 state are unrelated to electron dynamics. They reflect the large vibrational excess energy in the S0 state after internal conversion.

In fact, the oscillations in the electronic state give rise to the oscillations in the nuclear motion and the motion of the nuclei induces the electronic dynamics.

We have amended the text accordingly [Pg 10]

(v) The conclusion that ISC is much faster in acrolein than in ketene is rather trivial. This follows from the El Sayed rules without any dynamics simulations.

Indeed, that is what we wanted to show as a test of concept for our implementation. The ketene is the more interesting system with the nearby singlet/singlet crossing and the effect of ISC in accelerating the energy transfer needed to get there

We have further stressed this point in the conclusions [Pg 12]

This work does not provide new physical insights and cannot be recommended for publication.

We hope that we have convinced the referee otherwise

Reviewer: 2

The manuscript “Coherent mixing of singlet and triplet states in acrolein and ketene: A computational strategy for simulating the electron-nuclear dynamics of intersystem crossing (ISC)” by Danilov et al. studies the effect of spin-orbit coupling on the excited-state dynamics in acrolein and ketene.

The main message of this work is how the coherent mixing of singlet and triplet states affects the nuclear dynamics, via the average time-dependent Ehrenfest potential, and, in turn, the slow population transfer from the initially populated singlet to the triplet. In particular, in ketene, the spin-orbit coupling is very small compared to acrolein (at the S1 minimum where the trajectories are initialized). Nonetheless, the authors observe a strong effect on the dynamics of ketene, since the behavior of the system at the S0/S1 conical intersection is different when simulations w/o spin-orbit coupling are compared.

I find the manuscript well written and the message quite clear. I would recommend it for publication in The Journal of Physical Chemistry Letters, even though I have some general concerns with the employed methodology itself.

It seems to me that the authors make quite strongly the point that it is the Ehrenfest method, along with its average potential driving the nuclear dynamics, that yields the observed effects (for instance, modification of the S0/S1 dynamics in ketene upon inclusion of spin-orbit coupling). Surface hopping might give different results because the nuclear dynamics is propagated either on singlet potentials or on triplet potentials. Therefore, what is correct? I am not so sure that the average potential is the correct answer.

The most important point about Ehrenfest – when faithfully implemented, it yields not an average potential, but rather includes all the inter-state couplings in both the energy and its derivatives (gradient and hessian). So yes, the results should be different – that is one of the key points of this work. The nature of the potential is discussed in the paper.

We have also discussed this in our reply to the first referee.

We’ve added an extra sentence to methodological discussion further emphasise this point [Pg 6]

For attosecond dynamics, Ehrenfest might be a good approximation. However, for long-time dynamics, I would expect decoherence effects to be important and, thus, a surface hopping treatment, with proper decoherence corrections, might yield results closer to the “chemical reality” (see, for instance, J. Chem. Phys. 154 (2021) 094310 by the Truhlar group). I would like to ask the authors (1) if they could comment on the different dynamics that they would expect with a surface-hopping-like approach (AIMS would probably be similar and the exact factorization would yield something in between), and (2) why they think that their Ehrenfest dynamics describes better the reality of acrolein and ketene dynamics.

One might expect the surface hop method to yield reasonable results for acrolein – but much less so for ketene. We base this prediction on the fact that SOC (and triplet population) is so small in ketene that a majority of trajectories would simply never make the hop onto the triplet – hence never experience the extra excitation of the S0/S1 GDV normal mode that leads to the faster arrival at the singlet/singlet seam.

We have added a sentence to the conclusions to emphasise this point [Pg 12]

Another point that I would like the authors to comment on is the following. They report that the spin-orbit coupling at the S1 minimum, where the trajectory is initialized, in ketene is much smaller than acrolein. What about along the geometries visited by the trajectories? Do the authors use a constant value of the spin-orbit coupling?

In fact, the spin orbit coupling is re-computed at each step of the trajectory.

We added a sentence to address this in the main text and the SI [Pg 6 + S1]

Is it possible to know if this is a good assumption by computing the spin-orbit coupling at some additional geometries?

As we have pointed out in the previous comment, we compute the SOC at every geometry visited by the trajectory, for all possible states

Further, we refer the referee to the SI where we have added the SOC values for the points where we have also computed relativistic states [SI8]

Reviewer: 3

In this letter, the authors address a very interesting issue, namely the question whether spin–orbit mixed Born-Oppenheimer states are favorably used as starting points for a nuclear dynamics simulation of intersystem crossing processes. To this end, they set up an Ehrenfest dynamics scheme within the CAS-CI formalism, using Slater determinants with spin magnetic quantum number of MS=0 that can represent singlet as well as triplet states. Multiplicity mixing is achieved by inclusion of an empirical one-electron spin–orbit operator.

The performance of the method is examined for two example molecules, i.e., acrolein and ketene, for which they carry out dynamics simulations with and without spin–orbit coupling

and compare the results. The theoretical approach appears very promising for handling fast intersystem crossing processes, but there are still some open questions. Moreover, the reviewer has significant difficulties with the application part of the letter.

1) In the supporting information (page S2), the authors state that Hso is Hermitian and the SOC values are real. While the first statement is true, indeed, the second is not in general. Matrix elements of Hso typically are complex-valued. Can the authors describe in more detail why the SOC values are supposed to be real in their particular examples?

Hso is Hermitian but may have complex values. We take the magnitude of SOC, which is real. This is an approximation. We are interested in magnitude of SOC, to be used in the propagation only.

We have corrected the text and SI to emphasise this point [Pg 6 + S1]

2) In molecules with small SOC values, huge non-adiabatic couplings between the spin–orbit coupled states are expected to arise in the neighbourhood of a singlet–triplet crossing. How are these handled in the proposed approach?

The unique feature of our approach is that we use the full expression of gradient by including the coupled-perturbed equations, so that all the non-adiabatic couplings are included in the dynamics. Indeed, the effects seen in ketene are a manifestation of these couplings.

This is already emphasised in the theoretical part of the paper, but we have now re-iterated this point [Pg 6]

3) In the acrolein case, the authors examine the intersystem crossing between the S1 and T2 states which is an El-Sayed allowed process. They interpret their results in such a way that many re-crossings of the seam are required to reach a significant population of the triplet. Can they deduce a rate constant from their simulations?

We have now clarified this in the paper. The rate of triplet formation is not a meaningful quantity in these calculations because the result is a mixed state. Rather, one might say, for example in Acrolein, that after 500fs a molecule is 50% triplet and 50% singlet.

We have now discussed this explicitly in the introduction [Pg 3]

And how realistic is a multiple crossing back and forth between T2 and S1 given that the internal conversion from T2 to T1 presumably is competitive. Can the authors include this decay process in their simulations to get a more realistic picture?

Is the internal conversion from T2 to T1 competitive? This effect is included in our calculations, but we do not see this effect, presumably we are simply far away from the T1/T2 seam so the coupling is small.

We have added a brief comment to the text to address this point [Pg 9].

4) There is a severe problem with the ketene case. In the text and in Scheme 2b), the authors designate its S1 and T1 states as π-π* states, but their dominant configurations in Scheme 1 rather point towards a π-[illegible]* excitation. The occupation of the [illegible]* orbital appears to be the reason why the nuclear dynamics lead to a S1-S0 conical intersection. However, neither π-π* nor π-[illegible]* characteristics represent the electronic structures of the S1 and T1 states correctly. According to Szalay et al. (P. G. Szalay; A. G. Császár; L. Nemes. J. Chem. Phys. 105, 1034 (1996) https://doi.org/10.1063/1.471948 and references therein), the S1 and T1 states of ketene rather originate from an n-π* excitation. This gives rise to the question whether the ketene results presented in this letter are meaningful at all.

The issue here is one of chemical notation and orbital optimisation. A priori, we know what the active space for ketene must be. Ketene has 2 perpendicular pi systems; 2 $\pi_{||}$ orbitals and 3 $\pi_{\perp}$ orbitals and 6 electrons so the calculation requires a CAS(6,5) active space. In ketene the labels S0, S1 and T1 subscripts refer simply to the state ordering at the starting geometry (S_1 minimum). In CASSCF we choose the active space and then the states (S0, T1 and S1) correspond to the eigenvalues and eigenvectors of the CI. These orbitals will change in detail according to which state is being optimised. In other approaches, particularly those using a closed shell reference the orbital optimisation that is included in CASSCF is seen in CI mixing in the calculation using SCF orbitals. In practice the orbitals can change significantly during the optimisation process, so it becomes difficult to compare calculations [for example the EOM-CCSD paper mentioned] where the orbitals have been fully optimised with those using SCF orbitals. The confusion arises as to whether you regard the $\pi_{||}$ as an n orbital or a $\pi_{||}$ orbital. Furthermore, the states will re-order as the C-C-O bends (rendering the C2v linear C-C-O geometry symmetry labels inapplicable).

In terms of state ordering and notation our work agrees with the recent work of Xiao et. al. (JPhysChemA 117 32 7001-7008 (2013) doi.org/ 10.1021/jp312719a) which describes the lowest 3 singlets and 3 triplets, with their work characterising S1 and T1 as $\pi_{\perp} \rightarrow \pi_{||}{}^{*}$ states.

We have addressed this ambiguity in the caption for Scheme 1 [Pg 4]

Moreover, the potential energy surfaces of a $^{3}\pi$-π^{*} and $^{1}\pi$-π^{*} pair of states with equal spatial orbital occupation ought to be separated by a substantial energy gap. How can it happen that their potential energy surfaces cross as suggested in Scheme 2b?

The bonding force field for the singlet and triplet is completely different. In changing the C-O bond length, is repulsive in the triplet and attractive in the singlet. At some geometry, these states must cross – in fact this is the gradient difference vector. In conducting this work, we have plotted potential energy surface slices – and the S1/T1 crossing is there (for NM7 – the S1/T1 GDV)

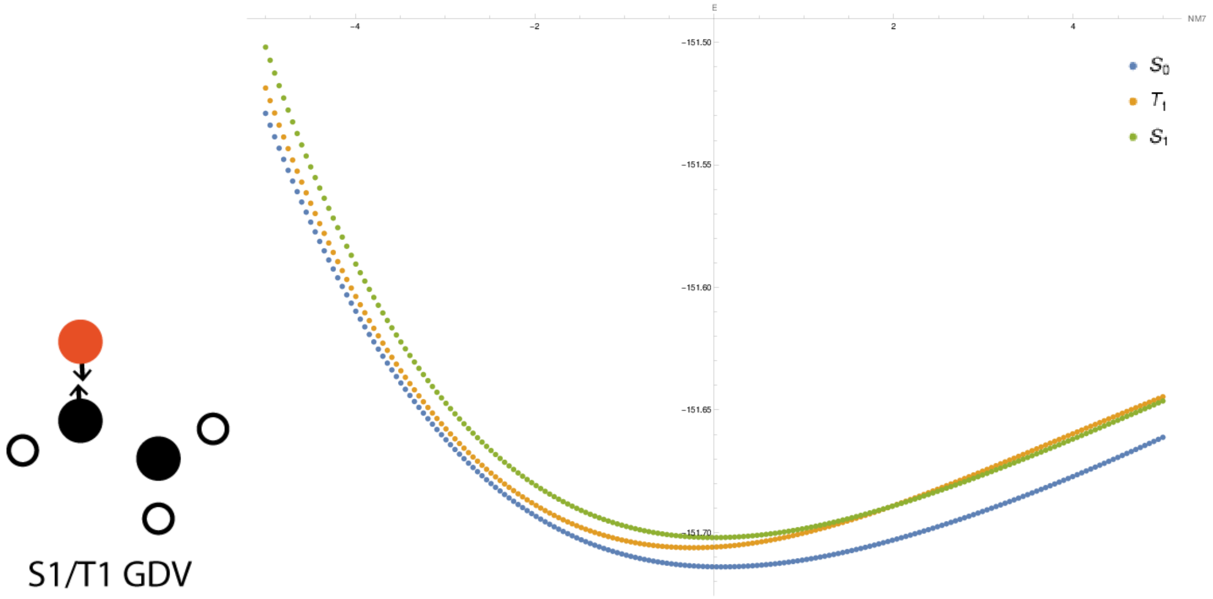

Please also note that we are not the first to identify a $^{1}(\pi \rightarrow \pi^*)$ / $^{3}(\pi \rightarrow \pi^*)$ crossing in ketene – the aforementioned paper of Xiao has identified minima along the same singlet triplet seams of crossing as well as Cui & Morokuma (JChemPhys 107 13 4951-4959 (1997) doi.org/10.1063/1.474890).

The above picture is already included in the paper as Scheme 2b and the reference are there as well.

5) Scheme 1: Why do the authors indicate a spin magnetic quantum number of MS=1 for the triplet states although they employ the MS=0 component in their calculations?

Yes, we use the Ms=0 state for dynamics but unfortunately, we cannot easily illustrate the Ms=0 triplet (of form $\frac{1}{\sqrt{2}}(|\alpha\beta\rangle - |\beta\alpha\rangle)$) in the scheme.

We have added a comment to the text to make this point clear. [Pg 4]

6) Please define TDSE.

This was an omission on our part and is now corrected [Pg 2]

jz-2023-01187t.R2

Name: Peer Review Information for "Coherent Mixing of Singlet and Triplet States in Acrolein and Ketene: A Computational Strategy for Simulating the Electron-Nuclear Dynamics of Intersystem Crossing"

Second Round of Reviewer Comments

Reviewer: 1

Comments to the Author

Despite a long rebuttal letter, the authors did not address the two main objections of this reviewer and did not make significant changes of the manuscript.

(1) The propagation of the (single) trajectory on the mean potential energy surface is a feature of the Ehrenfest approximation by construction. There is no need to demonstrate this for the special case of singlet-triplet spin-orbit coupling.

(2) As for all classical path methods, which approximate quantum wave packets by swarms of classical trajectories, stochastic sampling from a physically meaningful classical distribution corresponding to a quantum mechanical initial condition is necessary. Putting 1 eV of kinetic energy into a single GDV normal mode (!) is an unphysical initial condition and cannot provide quantitative results. Comparison of these simulation results with experiment for acrolein and ketene is not meaningful.

In the opinion of this reviewer, this work does not provide new physical insight. On the contrary, it rather confuses well-established concepts. The manuscript cannot be recommended for publication in a journal of Physical Chemistry, not to speak of JPCLett.

Author's Response to Peer Review Comments:

We note that you will be happy to accept our paper, but have asked us to take a look at the recent referee comments below, and address them in some form.

We have written a rebuttal and modified the paper.

The issue would appear to be trying to distinguish between the calculation aimed at elucidating a mechanistic effect (i.e. exploratory) as discussed in our paper and an exhaustive dynamics study which might aim to produce a result that might be directly comparable to experiment (such as a rate or a yield). In the section on the reaction path we have now tried to make this distinction completely clear.

We also noticed a typo in Scheme 2 – that’s now corrected.

We hope the paper can now be accepted.

I will be happy to accept your paper, but please, do take a look at the most recent round of the referee comments below, and perhaps, address them in some form. Thank you.

We have written a rebuttal and modified the paper [Pg 3]

The issue would appear to be trying to distinguish between a calculation aimed at elucidating a mechanistic effect (i.e. exploratory) as discussed in our paper and an exhaustive dynamics study which aims to produce a result which might be directly comparable to experiment (such as a rate or a yield). In the section on the reaction path [Pg 3] we have now tried to make this distinction completely clear.

Comments:

Despite a long rebuttal letter, the authors did not address the two main objections of this reviewer and did not make significant changes of the manuscript.

We tried to make those changes within the space limitations to emphasise the point that the focus of this paper is mechanistic

(1) The propagation of the (single) trajectory on the mean potential energy surface is a feature of the Ehrenfest approximation by construction. There is no need to demonstrate this for the special case of singlet-triplet spin-orbit coupling.

The referee is saying that an Ehrenfest computation in a basis of singlets and triplets with the inclusion of SOC will yield a mixed state. Of course we agree, however the unique nature of our work is the mechanistic interpretation of this mixing and the consequent geometry changes. Thus, the purpose of the paper is not to demonstrate the mixing per se, but rather to illustrate its effects (i.e. to explore the mechanistic implications).

(2) As for all classical path methods, which approximate quantum wave packets by swarms of classical trajectories, stochastic sampling from a physically meaningful classical distribution corresponding to a quantum mechanical initial condition is necessary. Putting 1 eV of kinetic energy into a single GDV normal mode (!) is an unphysical initial condition and cannot provide quantitative results. Comparison of these simulation results with experiment for acrolein and ketene is not meaningful.

As we have just stated, the purpose of our calculation is exploratory & mechanistic. We would certainly expect that if we wanted to produce quantitative results (such as a rate or yield) sampling would be required. We had already added a discussion of this in the last paragraph of the conclusions [Pg 12]

In the opinion of this reviewer, this work does not provide new physical insight. On the contrary, it rather confuses well-established concepts. The manuscript cannot be recommended for publication in a journal of Physical Chemistry, not to speak of JPCLett.

We have changed the discussion [Pg 3] to make clear the mechanistic and exploratory nature of our computations. The relevant paragraph is reproduced below;

Our focus in this paper is the mechanism of ISC on a mixed state. We will take an exploratory approach that is similar to the reaction path idea that is central to chemistry. Our computations are intended to explore the singlet/triplet crossing region of the potential energy surface in a similar way that one may explore the transition region of a thermal reaction. We now elaborate on this idea briefly.

We start from the fact that passing through a singlet/triplet crossing is a relatively rare event (like passing through a transition state[47–51]). In this way we can draw an analogy with the reaction path idea[52] that is central to mechanistic chemistry[53]. The reaction path approach is most commonly used with transition states[53,54]. It can be realised in dynamics (a dynamical reaction path) by starting a trajectory with the initial momentum in the direction of negative curvature[48,55]. Alternatively, one could start a trajectory at the 'reactants', with an initial momentum in the direction of the transition state. The results presented in this work are an excited state analogue of this technique; we start from an excited singlet minimum (our 'reactants') (labelled S_1 in both systems) with activation of the vibrational normal mode that corresponds to motion towards the S/T crossing seam (our 'transition state') – i.e. the normal mode approximately parallel to the gradient difference vector at the minimum energy point on the crossing seam (Figure 1). The essential difference, as we shall show subsequently, is that when the trajectory reaches the crossing seam, it does not proceed to 'products' but rather results in a mixture of singlet and triplet states, and after reaching a 'turning point' proceeds backwards across the seam again. This occurs many times with the population of triplet increasing on each passage of the crossing seam. Thus, we believe that our single 'reaction path' trajectory yields some mechanistic insight of ISC.
